# Patterns of bacterial motility in microfluidics-confining environments

**DOI:** 10.1073/pnas.2013925118

**Published:** 2021-04-19

**Authors:** Viola Tokárová, Ayyappasamy Sudalaiyadum Perumal, Monalisha Nayak, Henry Shum, Ondřej Kašpar, Kavya Rajendran, Mahmood Mohammadi, Charles Tremblay, Eamonn A. Gaffney, Sylvain Martel, Dan V. Nicolau, Dan V. Nicolau

**Affiliations:** ^a^Faculty of Engineering, Department of Bioengineering, McGill University, Montreal, QC H3A 0C3, Canada;; ^b^Department of Chemical Engineering, University of Chemistry and Technology, Prague, 166 28 Prague, Czech Republic;; ^c^Department of Applied Mathematics, University of Waterloo, Waterloo, ON N2L 3G1, Canada;; ^d^Department of Computer Engineering, École Polytechnique de Montréal, Montreal, QC H3T 1J4, Canada;; ^e^School of Mathematical Sciences, Queensland University of Technology, Brisbane, QLD 4000 Australia

**Keywords:** bacterial motility, microfluidic devices, space partitioning, wall escaper, wall accumulator

## Abstract

Understanding the motility behavior of bacteria in confining microenvironments, in which they search for available physical space and move in response to stimuli, is important for environmental, food industry, and biomedical applications. We studied the motility of five bacterial species with various sizes and flagellar architectures (*Vibrio natriegens*, *Magnetococcus marinus*, *Pseudomonas putida*, *Vibrio fischeri*, and *Escherichia coli*) in microfluidic environments presenting various levels of confinement and geometrical complexity, in the absence of external flow and concentration gradients. When the confinement is moderate, such as in quasi-open spaces with only one limiting wall, and in wide channels, the motility behavior of bacteria with complex flagellar architectures approximately follows the hydrodynamics-based predictions developed for simple monotrichous bacteria. Specifically, *V. natriegens* and *V. fischeri* moved parallel to the wall and *P. putida* and *E. coli* presented a stable movement parallel to the wall but with incidental wall escape events, while *M. marinus* exhibited frequent flipping between wall accumulator and wall escaper regimes. Conversely, in tighter confining environments, the motility is governed by the steric interactions between bacteria and the surrounding walls. In mesoscale regions, where the impacts of hydrodynamics and steric interactions overlap, these mechanisms can either push bacteria in the same directions in linear channels, leading to smooth bacterial movement, or they could be oppositional (e.g., in mesoscale-sized meandered channels), leading to chaotic movement and subsequent bacterial trapping. The study provides a methodological template for the design of microfluidic devices for single-cell genomic screening, bacterial entrapment for diagnostics, or biocomputation.

## Significance

Understanding bacterial movement is crucial for health, agriculture, environment, and industry. Studying the motility of five bacterial species in microfluidic environments showed that bacterial motility behavior is the result of a “tug-of-war” between hydrodynamics and local nanomechanics. In less confining spaces, bacterial motility is governed by hydrodynamics and can be approximately predicted by modeling developed for the simplest species. Conversely, in tightly confining environments, movement is mainly controlled by the steric interactions between flagella and the surrounding walls. Intriguingly, in mesoscale-sized geometries, hydrodynamics and bacterium–wall interactions overlap, either “constructively,” leading to smooth movement in straight channels, or “destructively,” leading to trapping. Our study provides a methodological template for the development of devices for single-cell genomics, diagnostics, or biocomputation.

Many motile bacteria live in confining microenvironments (e.g., animal or plant tissue, soil, waste, granulated, and porous materials) and consequently are important to many applications like health [infectious diseases (1, 2), pharmaceuticals (3), and nutrition (4)], agriculture [veterinary (5) and crops (6)], environmental science [photosynthesis (7), biodegradation (8), and bioremediation (9)], and industrial activities [mining (10) and biofouling (11)]. Bacterial motility is essential in the search for available physical space as well as for enabling bacterial taxis in response to external stimuli, such as temperature (12), chemical gradients (13, 14), mechanical cues (15), or magnetic fields (16).

To thrive in environments with diverse geometrical and physical characteristics, from open spaces to constraining environments, motile bacteria have evolved a multitude of propelling mechanisms (17), with flagellum-driven being the most common (18, 19). Flagellum-based machinery features various numbers of flagella (20) and designs: monotrichous, lophotrichous, amphitrichous, or peritrichous. The mechanics of this machinery, coupled with cell morphology (21) (e.g., coccus, rod-like, or curved) translates into several motility modes (e.g., turn angle, run-and-tumble, or run-and-flick) (22), and various motility behaviors (e.g., swimming, tumbling, and swarming) (17, 23). Environmental factors (24, 25) (e.g., chemical composition, viscosity, temperature, pH, and the chemistry and the roughness of adjacent surfaces) also influence bacterial motility.

“Pure” bacterial motility, unbiased by chemotaxis or fluid flow, was reported near simple flat surfaces (26, 27) and in channels (28–30). Simulations of model bacteria in analogous conditions were also undertaken (31–37), but owing to the complexity of bacterial mechanics (38), modeling from first principles did not provide sufficient understanding to accurately predict movement patterns of different species in complex, confined environments. Consequently, studies of the effects of bacterial geometry in confined geometries were limited to models of simple, monotrichous bacteria with an assumed rigid flagellum (32, 39).

Microfluidic devices (40, 41) are commonly used for the manipulation of individual or small populations of cells in micrometer-sized channels for medical diagnostics (42), drug screening (43), cell separation (44, 45), detection and sorting (46), and single-cell genomics (47). While microfluidic structures are used for the study of the motility of mammalian cells (48, 49), and microorganisms [e.g., fungi (50, 51), algae (52), or bacteria (29, 53–56)], these studies typically focus on a single species.

To make progress toward a more general understanding of the motility of individual bacterial cells in confining microenvironments, as well as to assess the extent to which the behavior of bacteria with complex architectures can be assimilated with that of the more predictable monotrichous bacteria, the present work investigated the movement of five species (i.e., *Vibrio natriegens*, *Magnetococcus marinus*, *Pseudomonas putida, Vibrio fischeri*, and *Escherichia coli*) in microfluidic geometries with various levels of confinement and geometrical complexity.

## Results and Discussion

**Fig. 1.**
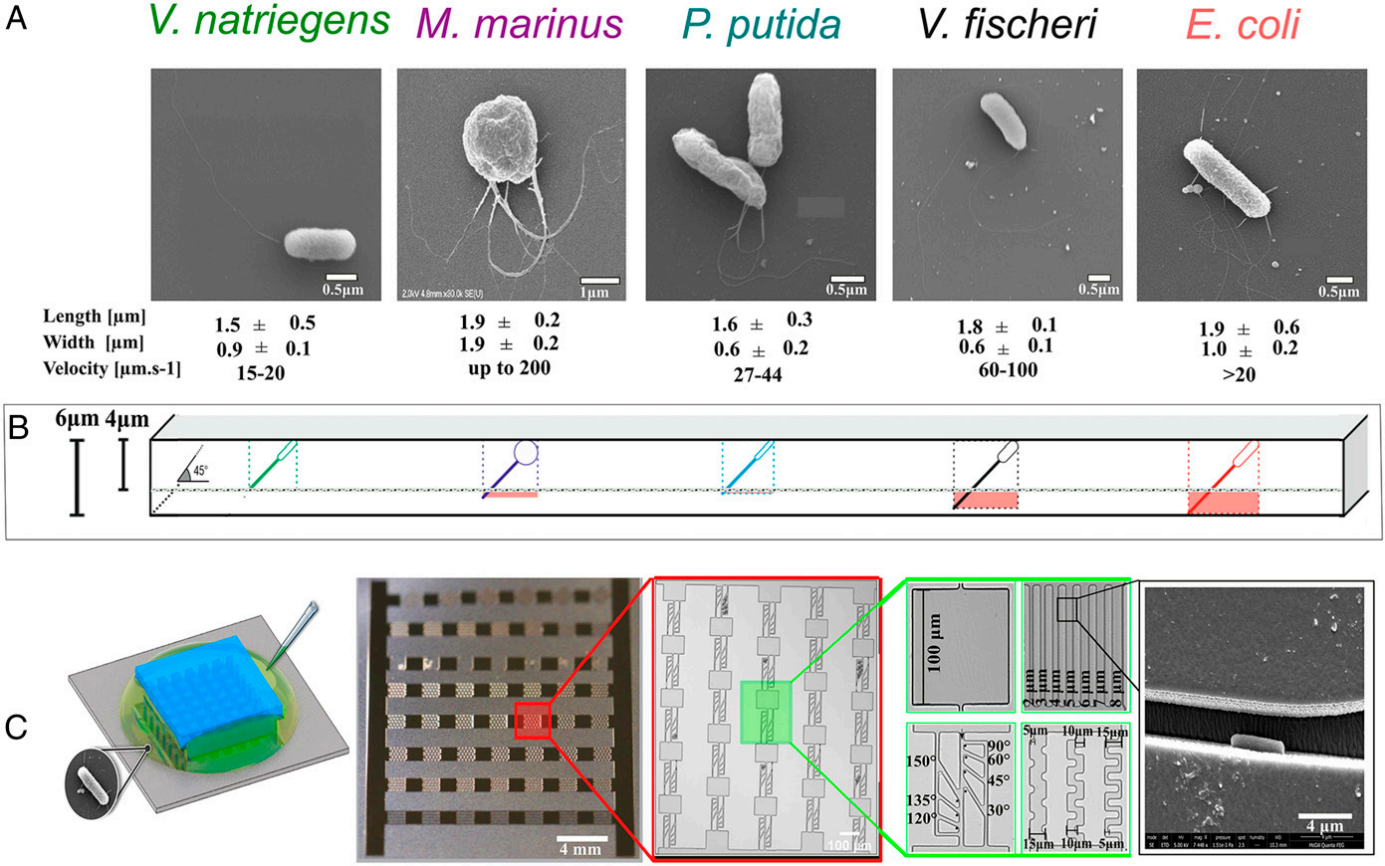
Microfluidics chip for testing microbial motility. (*A*) Scanning electon micrographs (SEM) images of bacteria studied with various architectures and dimensions (full details in *SI Appendix*, Table S1). (*B*) Graphical projection of the fit of the total bacterial length (body plus flagella) positioned at 45° versus the height of the microfluidic structures for 6 µm and 4 µm heights. (*C*) Sequential, from left to right, zoom-in images of the experimental device: 1) the bacterial suspension is introduced from the side of the chip attached to the cover slide; 2) the overall architecture of the chip; 3) zoom-in of one lane of experimental structures (sequence of angled channels separated by plazas); 4) detailed image of the experimental structures used in this study (i.e., plazas) and linear channels (top row), angled, and meandered channels (bottom row); and 5) SEM image of a bacterium (here, *E. coli*) in a channel.

The modulation of motility behavior by confinement was assessed by observing, by three-dimensional (3D) imaging, the movement of individual bacteria, presenting various characteristics (Fig. 1*A* and *SI Appendix*, Fig. S1) in microfluidic structures with high (6 µm) or low (4 µm) ceilings (Fig. 1*B*) and with various geometries (Fig. 1*C* and *SI Appendix*, Fig. S2) as follows: 1) large chambers with quasi-open spaces (“plazas”), 2) linear channels with various widths, 3) channels presenting lateral exits at various angles, and 4) meandered channels with various widths. In the absence of pressure and concentration gradients, this approach allowed the study of the interaction between hydrodynamics and the steric interactions of bacteria with the walls, unobscured by other external factors (e.g., rheo- and chemotaxis). Experimental, image analysis, and simulation protocols are fully described in *SI Appendix*.

### Motility in Large Chambers.

#### Impact of the distance between horizontal planes.

To minimize the possible coupling of the impact of horizontal planes, the designs of microfluidic chambers, made of polydimethylsiloxane (PDMS), had to find a compromise between their height and fabrication and operation issues. From the design perspective, it was found that a height of 6 µm (Fig. 1*A* and *SI Appendix*, Table S1) allows, conservatively, the unencumbered bacterial motility. Furthermore, preliminary experiments comparing motility in both types of microfluidic structures presented evidence (Movie S1) of the coupling of the impact on both horizontal planes on bacterial motility for those with 4 µm heights. Consequently, 6 µm–tall microfluidic structures were used for all further experiments. A detailed discussion is presented in *SI Appendix*.

#### Spatial distribution of bacteria.

**Fig. 2.**
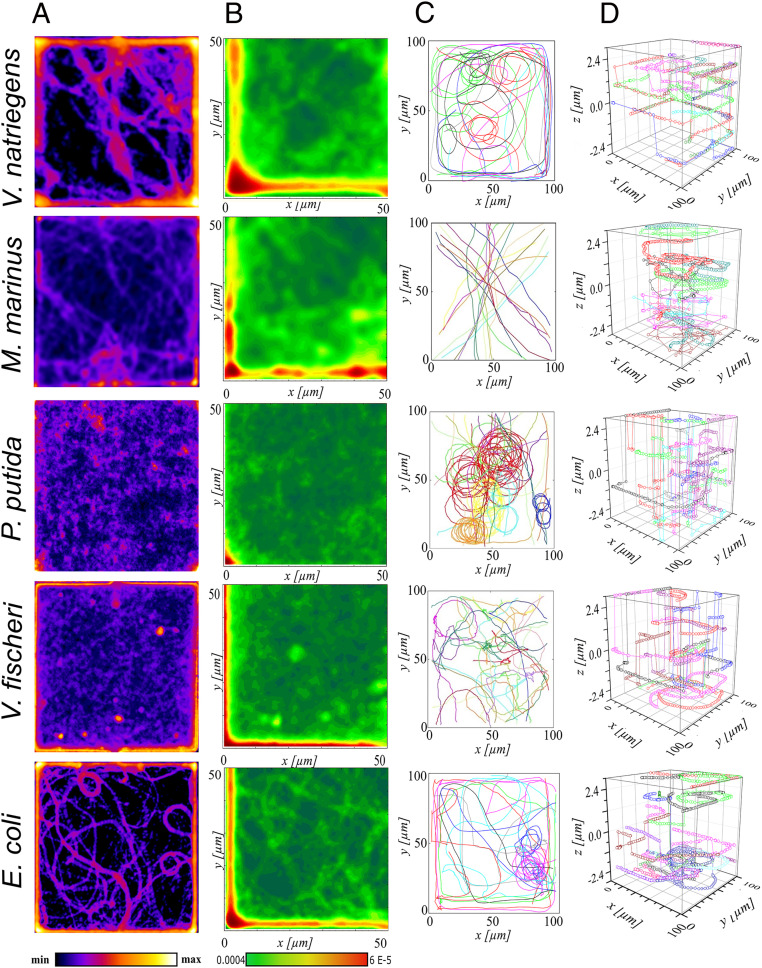
Motility in plazas with 6 µm high ceilings. (*A*) Density maps of bacterial locations. Color code (bottom): “min” and “max” represent no and the highest presence of bacteria, respectively. (*B*) Spatial distribution of bacteria obtained by superimposing and averaging the data from all four quarters of the density maps in *A*. Color code (bottom): frequency of bacterial presence, with red for the highest and dark green for the minimum probability. (*C*) Characteristic long2D projections of bacterial trajectories. (*D*) 3D bacterial trajectories. By rows, from top to bottom, are the following: *V. natriegens* (average count of bacterial positions in each frame, *n* = 14/frame); *M. marinus* (*n* = 12/frame); *P. putida* (*n* = 15/frame); *V. fischeri* (*n* = 15/frame); and *E. coli* (*n* = 13/frame). Movie S1 presents the bacterial movement in plazas, with representative trajectories (similar to *C*).

The bacterial species studied presented different motility behaviors with respect to proximity of vertical walls and corners (Fig. 2 *A*–*C*). First, *V. fischeri*, *V. natriegens*, and *E. coli* moved at small distances from vertical walls. Second, *M. marinus* presented an uneven, broken density near vertical walls, due to the frequent “ping-pong”–like collisions and reflections (Movie S1). Third, *P. putida* presented an even spatial distribution throughout the chamber.

The 3D imaging and z-stack sectioning of bacterial trajectories in 6 µm–tall plazas (Fig. 2*D* and *SI Appendix*, Figs. S6–S8) revealed a similar behavior in the central area close to the horizonal walls (i.e., free of the possible edge effects from the vertical walls). *V. natriegens*, *V. fischeri*, and *E. coli* presented trajectories in proximity to—and parallel with—the horizontal walls. This was not the case for *P. putida* and *M. marinus*, which frequently fluctuated between z-planes (*SI Appendix*, Figs. S7 and S8). Statistical analysis of the bacterial positions (*SI Appendix*, Fig. S9) showed that *V. natriegens*, *V. fischeri*, and *E. coli* moved preferentially in a parallel plane to the horizontal walls and that *P. putida* and *M. marinus* presented a rather uniform distribution of positions on the vertical axis.

#### Theoretical classification of bacterial motility behavior.

For bacteria that are propelled by a flagellum or flagellar bundle behind the cell, the fluid flow generated by swimming has a dipolar structure: the fluid is pushed backward by the flagellum and pulled forward by the cell body. This flow has been shown to attract swimmers to solid walls, causing them to remain close to the wall for long time periods despite rotational Brownian motion (57). A separate effect of swimming near surfaces is that hydrodynamic interactions between the wall and rotating flagellum and between the wall and counter-rotating cell body, respectively, lead to bacteria swimming in circular orbits when they are close to a wall (58).

Detailed hydrodynamic modeling of monotrichous bacteria showed that the geometrical parameters of the cell (length and width) and of the helical flagellum (length, helical amplitude, and wavelength) determine the motility behavior near a single flat surface (32). Based on this modeling framework, correlated with the experimental observations from the present study, three classes of behavior were observed, depending on the geometry of the bacterium. “Wall accumulators” descend to the walls and exhibit a strong propensity for swimming in the closest vicinity to the wall (with a separation of tens of nanometers between the bacterium and the surface), where steric interactions are likely, thus making difficult the precise prediction of motility behavior even for the simplest monotrichous bacteria. When bacteria swim at distances further than this from the wall but at a nearly constant separation, exhibiting the characteristic circular orbits predicted by simpler analysis, they are classified as “stable swimmers parallel to the wall.” It was observed (26) that dynamical interactions are negligible before collisions with the walls, but once bacteria swim on parallel planes a few micrometers away from surfaces, hydrodynamic forces maintain long residence times in this region. Finally, when hydrodynamic interactions result in bacterial movement away from surfaces, they are classified as “wall escapers.” The demarcation between these classes is approximate, due to the inherent stochasticity of bacterial motility.

**Fig. 3.**
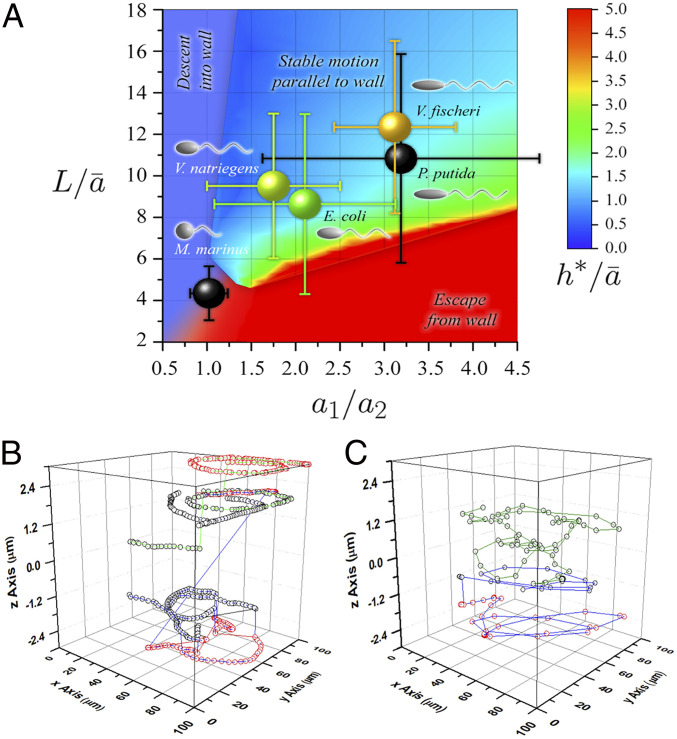
Prediction of motility behavior. (*A*) Bacterial positions, according to their dimensions, on a motility “map” (32), derived from hydrodynamic principles, for monotrichous bacteria. *V. natriegens*, *V. fischeri*, and *E. coli*, “swim parallel to walls” (confirmed experimentally, Fig. 2*B* and *SI Appendix*, Fig. S9). *M. marinus* is placed at the boundary between “wall accumulators” and “wall escapers” regions (confirmed experimentally by its wall-bouncing behavior). *P. putida*, with the largest variability of sizes, straddles the extreme “swimming parallel to wall” and “wall escaper” regions (confirmed by spatial distribution in Fig. 2*B* and *SI Appendix*, Fig. S9). The legend (updated from ref. 32, *SI Appendix*, Table S2) is as follows: a_1_ = polar radius of cell body (half the cell length); a_2_ = equatorial radius of cell body (half of the diameter diameter); [a_1_/a_2_] = aspect ratio of the cell body; L = curvilinear length of the flagellum (approximated by the axial length of the flagellum); ā = radius of sphere with volume of cell body; L/ā = nondimensional length of the flagellum/a; h* = optimal distance from wall (for swimmers parallel to walls); and h*/ā = nondimensional stable distance from wall. The colors of bacterial coordinates approximately replicate the color equivalent to h*/ā (determined from z-stack analysis). (*B*) Example of a bacterium moving stable parallel to the walls: *E. coli* (also exhibiting “escape from wall” jumps). (*C*) Example of a “wall escaper” bacterium: *M. marinus*.

Two key geometrical parameters determining whether a particular bacterium is an accumulator, escaper, or moving parallel to the wall are 1) the cell body aspect ratio and 2) the length of the flagellum. Higher aspect ratios (more rod-like) and shorter flagella encourage escape from walls (Fig. 3). For geometries at the boundary between parallel motion and escapers, it is possible for a bacterium to exhibit either stable motion close to the wall or escape depending on the angle of approach to the wall. It is useful to first determine the behavior of bacteria near a single wall because this is indicative of motility in more complex environments. For example, simulations showed that parallel–stable swimmers and escapers had different characteristics when placed between parallel walls (35) and in corners of rectangular channels (39). However, the variability of characteristic bacterial dimensions adds to the inherent stochasticity of movement. This in turn makes the demarcation between motility classes approximate. Details of the modeling used in Fig. 3 are given in *SI Appendix*, and the characteristic dimensions of bacteria are presented in *SI Appendix*, Table S2.

While these theoretical studies were based on a model with a single, polar flagellum, it was demonstrated that such models accurately reproduce the experimentally observed radius of curvature of near-wall tracks for *E. coli*, which swim with several flagella (31). Therefore, it is expected that this classification serves as a useful conceptual background for the characterization of motility behavior in relation to a solid surface, even though most of the species in the current study are architecturally more complex than the monotrichous model (here, *V. natriegens*). Indeed, the propensity to move near surfaces was observed experimentally for several nonmonotrichous bacterial species, for instance (extensively) for *E. coli* (26, 27, 30, 31, 57, 59), but also for *Serratia marcescens* (29) and *Pseudomonas aeruginosa* (60).

#### Comparison of experimental and theoretically predicted behavior.

By comparison with monotrichous model bacteria of equivalent dimensions, *M. marinus* is predicted to be a wall accumulator, but it is actually near the boundary between accumulators and escapers (Fig. 3). All other species are expected to maintain stable motion parallel to and near the walls (Fig. 3), although variability within populations is sufficient for some individuals to be classified as escapers. There are elements that correlate well with the predicted motility behavior of simple bacteria with that of the more complex geometries studied as well as explanations for the deviations from this general “motility landscape” (Fig. 3):

1) Our experiments showed that *M. marinus* did not exhibit stable motion parallel to the wall but rather a “ping-pong”–like movement, with abrupt approaches to the walls alternating with equally abrupt breakouts. Recently, a model of the movement of a polar biflagellate bacterium (61), based on *M. marinus*, showed that such wall escaping (scattering) behavior could occur for certain arrangements of the two flagella. Additionally, it was recently reported that *M. marinus* swims with one flagellar bundle in front of the cell body and one behind (62), a mode of motility that is fundamentally different from the monotrichous model.
2) The density maps, probability maps, long trajectories, represented as two-dimensional (2D) projections and in 3D (Fig. 2 *A*–*D*, respectively) for *P. putida* and *E. coli*, showed characteristics of both escapers, more apparent for *P. putida*, and movement parallel to the wall, more apparent for *E. coli.* The persistent circular orbits indicate motion close to the horizontal walls, and for *E. coli*, the long trajectories along the vertical walls also highlight boundary accumulation. In contrast, the long, relatively straight trajectories through the middle of the chamber and frequent transitions between z-planes represent wall escaping behaviors. These seemingly contradictory observations are, in fact, consistent with the variability found in the measured cell shapes and flagella lengths. While the average values for both *P. putida* and *E. coli* lie within the movement parallel to the wall regime (Fig. 3), the spread of parameters extends considerably into the wall escaper region.
3) Density and probability maps, as well as 2D projections and 3D bacterial trajectories (Fig. 2 *A*–*D*, respectively), are consistent with the placement of *V. natriegens* and *V. fischeri* deep in the movement parallel to the walls, according to the theoretical predictions in Fig. 3. Both species showed circular trajectories (more prominent in *V. natriegens*) and high densities around the perimeter of the chamber. Interestingly, *V. natriegens* was often observed swimming parallel to the vertical walls but at distances of around 3.5 µm from the wall (Fig. 2*B*) rather than keeping almost in contact with the wall. This type of parallel motion was found in simulations of boundary accumulators in corners of channels (39).

#### Motility patterns.

The longest trajectories of bacterial motility in plazas had characteristics that were the most species specific (Fig. 2*C* and Movie S1, top row). *V. natriegens*, *E. coli*, and *V. fischeri* presented, to various degrees, two classes of trajectories: 1) movement along the vertical and horizontal walls and, when detached, 2) circular motions, until again attaching to the walls. *M. marinus* exhibited a “ping-pong”–like motility pattern, generally following relatively straight paths until it approached and scattered off a vertical wall, resulting in a statistically higher density localized near the walls (due to frequent collisions). There was little discernible movement along the vertical or horizontal walls of the plaza, and no complete circular orbits were observed. Two classes of behavior were present in the longest trajectories of *P. putida*. Some were relatively straight, spanning from one side of the chamber to the other, whereas other trajectories were circular and persisted for many overlapping cycles. Long trajectories around the perimeter of the chamber, as observed for *V. natriegens*, *E. coli*, and even *V. fischeri*, were uncommon for *P. putida*.

#### Circular motion.

The circular motion of bacteria near surfaces was previously reported for *E. coli* both at air–liquid (27) and solid–liquid interfaces (58, 63) and for *P. putida* at solid–liquid interfaces (33, 64). Counterintuitively, despite their very different flagellar arrangements (Fig. 1*A* and *SI Appendix*, Fig. S1 and Table S1), circular patterns were also observed here for *P. putida*, to a lesser extent for *E. coli*, and for *V. natriegens* (Fig. 2 *C* and *D*). Theoretically, the hydrodynamic interactions between a flat surface and a bacterium swimming on a parallel plane to it are indeed able to explain this curved pattern of trajectories (58, 60).

In summary, in quasi-open spaces, such as plazas, when the movement is limited only by parallel vertical or horizontal walls placed at distances considerably larger than the size of bacteria, their motility can be approximately characterized as stable movement parallel to the wall, wall escapers, or—rarely—as wall accumulators, as derived from bacterial geometric parameters and hydrodynamics-based modeling of the movement near surfaces of monotrichous bacteria.

### Motility in Tightly Confining Geometries.

#### Motility in linear channels.

Following the experiments in plazas with high and low ceilings and to avoid (to the extent possible) the impact on motility from more than two vertical walls, further experiments used only microfluidic channels with a 6 µm distance between the horizontal planes.

#### Overall motility characteristics; sinusoidal movement.

**Fig. 4.**
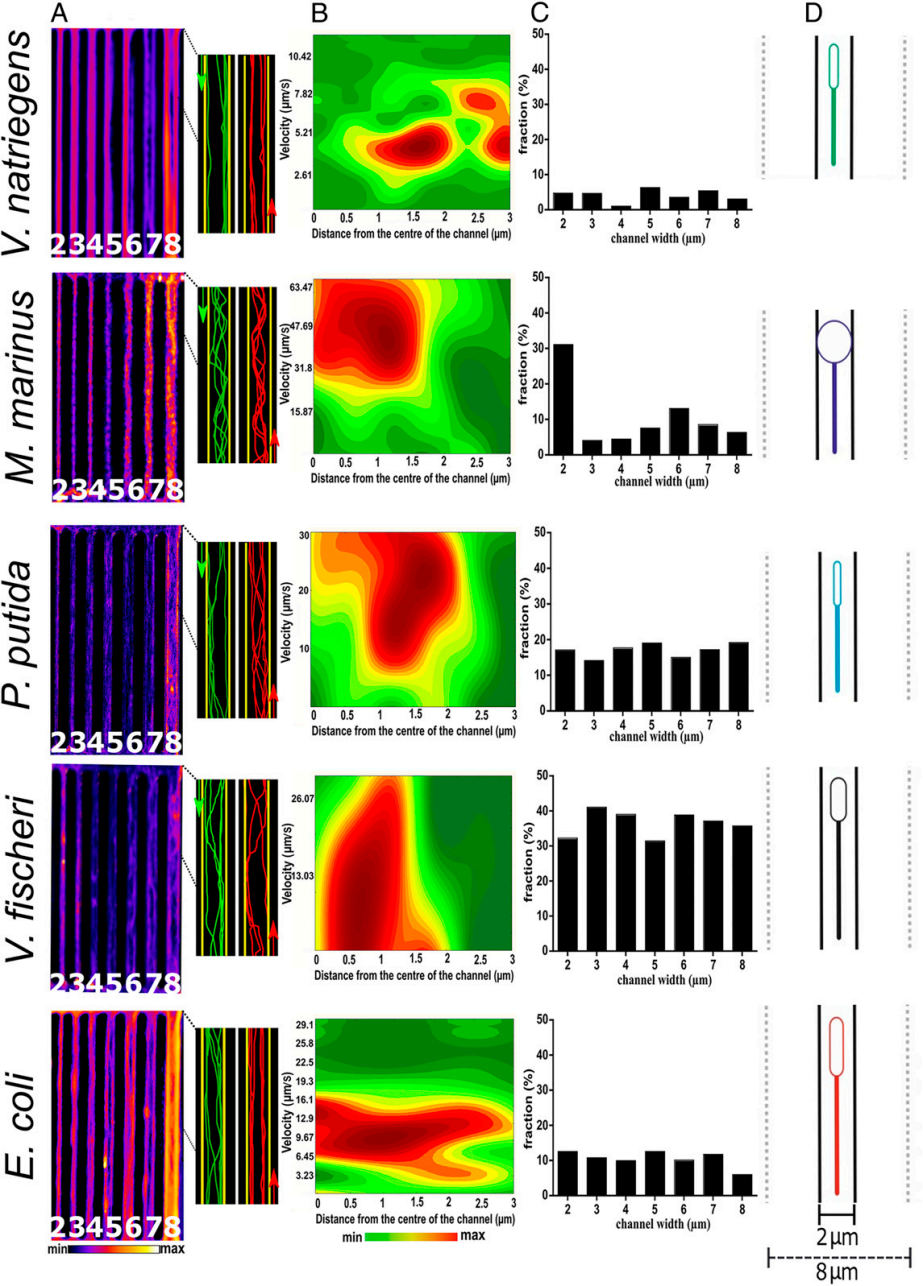
Bacterial motility in linear channels. (*A*, *Left*) Density maps of the movement patterns of bacteria in channels with different widths. (*A*, *Right*) Bacterial trajectories in 8 µm wide channels, moving from one direction (green) or from an opposite one (red). (*B*) Double histograms of velocity (*y*-axis) versus normalized distance from the center of the linear channel (channel wall on the extreme right) for 6 µm × 6 µm channels (the full analysis is presented in *SI Appendix*, Fig. S14). *E. coli* and *V. natriegens* present a specific bimodal distribution of velocities near the wall. (*C*) Influence of the channel width on the fraction of U-turns. By rows, from top to bottom, are the following: *V. natriegens* (average count of bacteria each frame, *n* = 20/frame); *M. marinus* (*n* = 10/frame); *P. putida* (*n* = 19/frame); *V. fischeri* (*n* = 18/frame); and *E. coli* (*n* = 22/frame). (*D*) Graphical representation of the top view of a bacterium with their average dimensions, in linear channels. The thick and dotted lines represent the minimum and maximum channel widths. Movie S2 presents bacterial movement in channels, with representative trajectories (similar to *A*, *Right*).

When laterally confined in wider channels (e.g., 6 to 8 µm), *V. natriegens* and *E. coli* showed the strongest propensity for moving along walls (Fig. 4*A* and *SI Appendix*, Fig. S10 for 3D trajectories), correlating well with their motility behavior in plazas (Fig. 2*A* and *B*) and their movement parallel to the vertical (Fig. 2*C* and *D*) and horizontal walls (*SI Appendix*, Fig. S9).

*P. putida* exhibited an apparent sinusoidal movement, especially in larger channels (Fig. 4*A*). A Fast Fourier Transform (FFT) analysis of the trajectories (*SI Appendix*, Figs. S11 and S12*B***)** indicated that *V. natriegens*, *V. fischeri*, and, to a much lesser extent, *E. coli* also present sinusoidal movement characteristics, with wavelengths increasing roughly proportionally with an increase in channel widths (*SI Appendix*, Fig. S12*B*). It was demonstrated (39, 65) that monotrichous wall escapers (with this behavior being predicted, partially, for *P. putida* in Fig. 3) move in distorted helical paths in channels of large rectangular transversal section. This upwards correlation between motility wavelengths and available volume for movement is similar to the larger radii of the circular movement in plazas with higher ceilings than in those with low ceilings (Fig. 2*C* and *SI Appendix*, Fig. S5*C*). *M. marinus* also exhibited sinusoidal-like behavior, but the FFT analysis showed that this movement is only the result of frequent collisions to, and bouncing from, the walls.

In narrower channels (i.e., 3 to 6 µm), the tighter confinement increasingly forced bacteria to move along the channel axis (except for *M. marinus*) rather than exhibited their motility behavior observed in open spaces (plazas). Moreover, in tighter (but still larger than the lateral size of the cell) channels, bacterial movement appeared to benefit from both hydrodynamics and steric interaction with the walls, which synergistically push bacteria in the same direction due to the lateral-only confinement of straight channels (66).

##### Velocities in channels.

Analysis of the velocities in straight channels appeared to further substantiate the synergy between hydrodynamics-driven and steric interactions–driven motility mechanisms. Indeed, while *M. marinus* exhibited a moderate decrease in average velocity with the decrease of the width of the channel, including compared with that in the plazas, due to an increase in collisions with the walls, all other species did not show any notable and systemic velocity variation with channel widths (*SI Appendix*, Fig. S13). Furthermore, the double histograms of the velocity in channels (Fig. 4*B*, for rectangular 6 × 6 µm channels; full analysis in *SI Appendix*, Fig. S14) revealed that *V. natriegens* and *E. coli* presented a distinctive bimodal distribution of velocities at the walls, with one velocity higher and one lower than the overall velocity. This bimodal distribution, for the species with the lowest ratios of the cell body and of the flagella (a_1_/a_2_ and L/ā, respectively, Fig. 3 and *SI Appendix*, Table S2), could be the result of separate instances of short-term cell adhesion to the wall and movement acceleration due to the steric interaction of flagella with the walls. In this context, it was reported (67) that the interaction between the walls and the flagella of *E. coli* translates into a “thrusting aid” for those bacteria running smoothly along solid surfaces. It was also reported, for *E. coli* (30, 68), *B. subtilis* (69), and *S. marcescens* (29), that bacteria exhibited higher velocities in narrower channels (which eventually decreases significantly in even narrower channels, due to the severe mechanical constraints applied to the cells), which is supported by the bimodal distribution of velocities observed for *E. coli* (and *V. natriegens*) here.

##### Straight versus U-turn movements.

In straight channels, bacterial motility was expected to be increasingly driven by steric interactions, to the detriment of hydrodynamics, with a decrease in channel widths. This increased impact of the steric interactions can explain the species-specific proportion of U-turns (Fig. 4*C*). First, the species with the lowest ratio of flagellum/length/cell body (i.e., *V. natriegens* and *E. coli*) (Fig. 3) had the lowest overall proportion of U-turns, with an apparent decrease of U-turns with the channel width for the larger *E. coli* (Fig. 4 *C*, *Bottom*). Conversely, the species clustered at higher characteristic values of L/ā and a_1_/a_2_ ratios (i.e., *P. putida* and *V. fischeri*) (Fig. 3) have a considerably higher proportion of U-turns than *V. natriegens* and *E. coli*, and there was even a considerably higher proportion for *V. fischeri* (Fig. 4*D*, fourth from the top). Second, *M. marinus*, with its characteristic frequent collisions and rebounds from the walls, had a low ratio of U-turns, with the notable exception of the 2 µm–wide channels. This unique behavior can be explained by the extreme steric interactions of *M. marinus* with both walls in channels with 2 µm widths, (i.e., as large as the cell body) (Fig. 4*D*, second from the top), resulting in the bacterial cell being “pinned” by both vertical walls then “flipped” in the 6 µm–tall vertical plane of the channel, followed by the movement in the opposite direction. Third, *P. putida*, experiencing intermittent wall contact, exhibited a similar ratio of U-turns as *V. natriegens* and *E. coli*. Fourth, *V. fischeri*, which swim the closest to the wall (Fig. 2*B*), had the highest ratio of U-turns.

In summary, these results demonstrate that, when a strong and complex coupling exists between the interaction by parallel walls placed at distances similar to the dimensions of bacteria, their motility is primarily governed by the local steric interactions between the walls and the flagella and, in extreme confinement, the cell body. Consequently, the increase in confinement with narrower channels leads to a decrease in hydrodynamics-based propulsion, and the dilution, or outright disappearance of the classes of motility behavior observed in open spaces.

#### Motility in channels with angled exits.

**Fig. 5.**
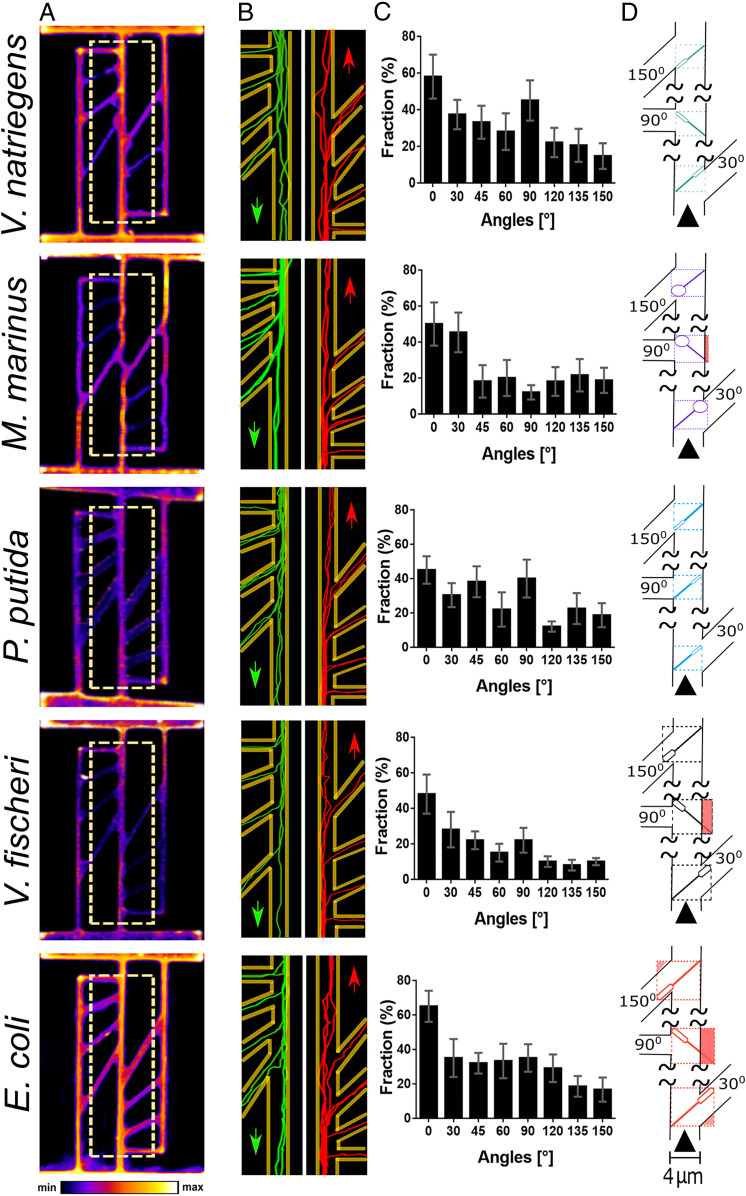
Bacterial motility in channels with angled exits. (*A*) Density maps of the movement patterns of bacteria in straight parallel channels, connected by side channels at angles ranging from 0° to 150°. All channels are nominally 4 µm wide. (*B*) Bacterial trajectories, either from top (green) or from the opposite direction (red). (*C*) Frequencies of bacteria moving at different exit angles. By rows, from top to bottom, are the following: *V. natriegens* (average count of bacteria each frame, *n* = 10/frame); *M. marinus* (*n* = 8/frame); *P. putida* (*n* = 10/frame); *V. fischeri* (*n* = 13/frame); and *E. coli* (*n* = 11/frame). (*D*) Graphical representation of the top view of bacterium with average dimensions in the angled channels (few representative angles). The areas in light brown represent spaces that exceed the dimensions of the respective bacteria in the respective position. Movie S3 presents bacterial movement in angled networks and representative trajectories (similar to *A*, *Right*).

In the structures with angled exits (Fig. 1*C*, lower right of the fourth image from the left), all bacterial species had a large preference for moving in straight trajectories along the middle axis of the channel, as qualitatively suggested by the density maps (Fig. 5*A*), by representative trajectories (Fig. 5*B* and Movie S3), and by representative bacterial 3D trajectories (*SI Appendix*, Fig. S15). Even for the smallest exit angle (i.e., 30°), the probability of movement in a straight line instead of exiting laterally (estimated as the ratio between bacteria moving straight and the total number that arrived at that intersection) ranges from 72% (for *P. putida*) to 58% (for *M. marinus*). While the general trend for all bacteria was that the exiting probability decreased with increasing exit angle, there were some species-specific details (Fig. 5*C*). First, *V. natriegens*, *E. coli*, and *P. putida* had a clearly decreasing exiting probability with an increase in exit angle, while for *V. fischeri* this trend was less visible, and *M. marinus* exhibited a rather indifferent relationship between exit probabilities and exit angles, following an abrupt drop at angles higher than 30°. Second, all species other than *M. marinus* had a relatively higher exiting probability at 90° angles.

This species-specific motility behavior in angled channels appeared to be the result of bacterial movement being driven by both local hydrodynamics and by steric interactions with the walls. First, the decrease of exit probabilities with exit angles for all species, but especially for *V. natriegens* and *E. coli*, resembles the lower frequency of turning by large angles in open spaces (*SI Appendix*, Fig. S3). The deflection angles in open spaces (*SI Appendix*, Figs. S3 and S4) are near-instantaneous measurements, and while longer integration times would lead to larger apparent values, this could also incorporate other sudden changes of direction, thus obscuring the inherent propensity of bacteria for sideways movement. With this qualification, it is reasonable to expect a connection between the propensity to escape laterally at set angles in angled channels (Fig. 5*C*) and the deflection angles in open spaces. However, this similarity had notable limitations (e.g., all species studied had negligible probabilities of deflection angles at much lower angles than those for bacteria in the angled channels). Second, while the wide spread of deflection angles in plazas (*SI Appendix*, Figs. S3 and S16) for *P. putida* could justify its relatively wide spread of exit probabilities in angled channels, *E. coli*, which had a narrow distribution of deflection angles, had a considerably larger and wider distribution of exit probabilities in angled channels than *P. putida*. Similarly, while both *V. natriegens* and *V. fischeri* exhibited a monotone decrease of frequency with increasing deflection angles in plazas (*SI Appendix*, Figs. S3 and S16), this behavior translated into a monotone decrease of exit probabilities in angled channels only for the former, whereas the latter did not show any obvious correlation between exit probabilities and respective escape angles. Finally, *M. marinus* had a monotone decrease of frequency with increasing deflection angles (after 10°) but an approximately flat relationship between the exit probabilities and escape angles (after 30°).

These observations suggest that, in addition to species-specific hydrodynamics-driven spread of deflection angles in open spaces (plazas), another mechanism was also responsible for determining the exit probabilities in angled channels. Indeed, the species that exhibited a notable departure from the expected extrapolation of behavior in open spaces is also the species whose dimensions exceed the clearance in the angled channels (i.e., *E. coli* and *V. fischeri*) (Fig. 5*D*). Conversely, the species whose dimensions did not surpass the clearance in the angled channels (i.e., *V. natriegens* and *P. putida*) are also those which exhibited a reasonable extrapolation of deflection angles in open spaces to a monotonical decrease of exit probabilities with escape angles. The frequent collisions and bouncing of *M. marinus* had the effect of leveling the exit probabilities regardless of the escape angles (except for 30°, for which there is enough turning space and therefore a higher exit probability, Fig. 5*D*).

It must be also noted that the confinement at the intersection between central and lateral channels did not fully correlate with the respective exit angle. For instance, the 150° exit offered the largest volume available for movement at the intersection between the axial and lateral channels (highlighted in *SI Appendix*, Fig. S2), thus making the comparison with U-turns (at 180°) in tight linear channels, with no variation of widths, inconsistent. Finally, the relatively higher escape probabilities for 90° angles could be the result of smaller free volume at the intersection of the axial channel, with steric interactions biasing bacteria toward lateral exits.

In conclusion, bacterial motility studies in angled channels revealed that when the level of confinement is low, due to the large volume at cross-intersection in relation to smaller bacterial sizes, the movement is mostly driven by hydrodynamics, as an extension of the behavior observed in open spaces. Conversely, when the confinement is tight, due to larger bacterial sizes, the local steric interactions between flagella and the walls contribute substantially to the motility behavior.

#### Motility in meandered channels.

The trapping of bacteria in purposefully designed microfluidics structures is of special interest to various applications [e.g., single-cell genomics (70) and accelerated evolution (71)], and therefore, the responsible mechanisms were studied (72, 73).

**Fig. 6.**
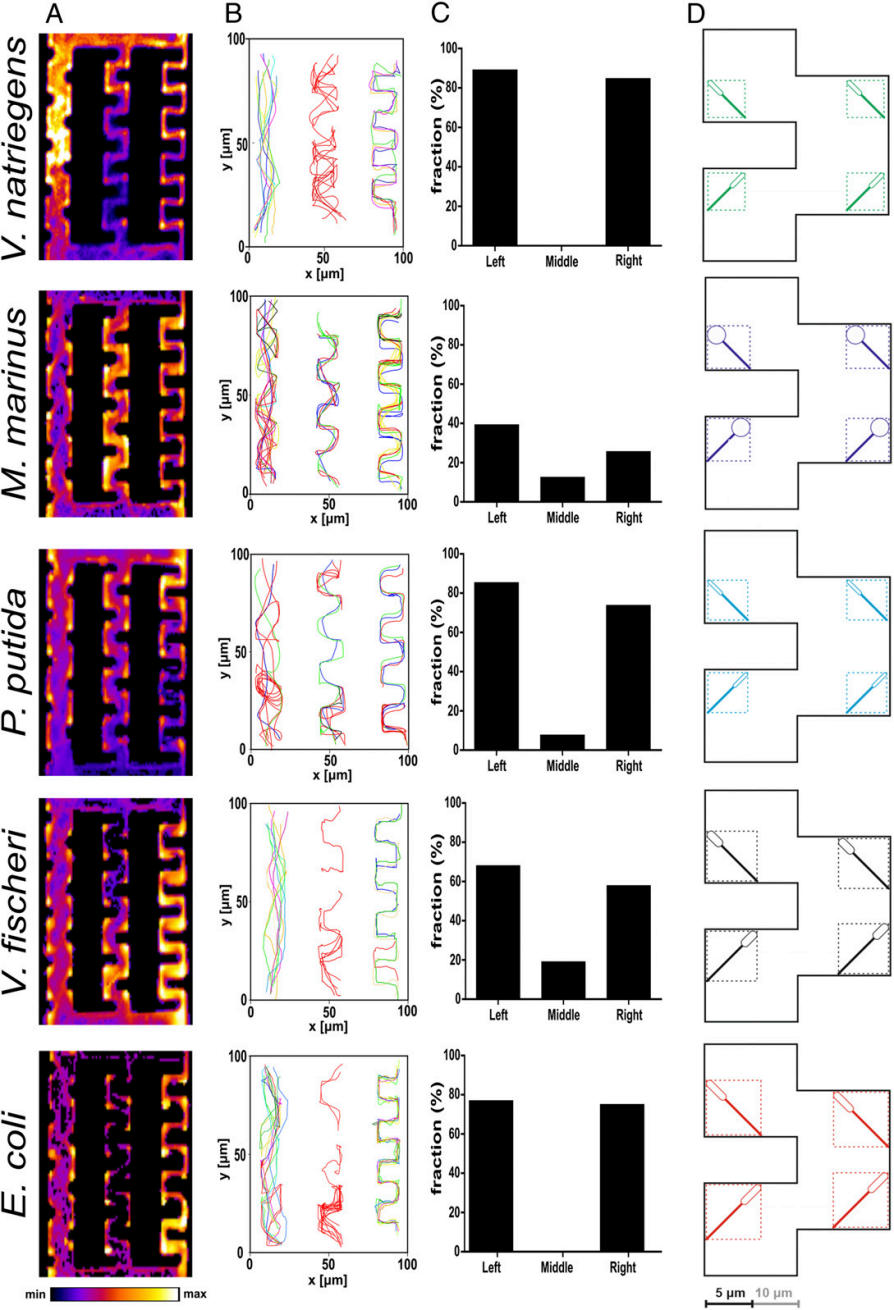
Bacterial motility in meandered channels. (*A*) Density maps of the bacterial movement patterns. (*B*) Representative tracks of the bacterial motility (trajectories in red are for bacteria that took U-turns or got trapped). (*C*) Frequency of bacteria making successful exits, relative to those that are trapped, or performed U-turns (unsuccessful tracks) for each meandered channel. By rows, from top to bottom, are the following: *V. natriegens* (average count of bacteria each frame, *n* = 18/frame); *M. marinus* (*n* = 12/frame); *P. putida* (*n* = 22/frame); *V. fischeri* (*n* = 25/frame); and *E. coli* (*n* = 19/frame). (*D*) Graphical representation of the top view of bacteria in the mesoscale-sized channel. Movie S4 presents bacterial movement in comb-like channels and representative trajectories (similar to *B*).

The meandered system comprised three channels, each with a different gap between the edge of the “teeth” (i.e., 5 µm [left], 10 µm [middle], and 15 µm [right]) (Fig. 1*C*, lower right, fourth image from left). The tightly confined, 5 µm–wide meandered channels made the motility of all species more complex (Fig. 6 and *SI Appendix*, Fig. S17 and Movie S4). The elastic-like collisions of *M. marinus* resulted in frequent trappings and, consequently, a considerably lower overall “success rate” (defined as the ratio of bacterial entries versus exits, at steady state) than the rest of the bacterial species (Fig. 6*C*). In addition, the 90°-angled corners appeared to operate as traps for *E. coli* and to a lesser extent for *V. natriegens* (bright spots in the density maps in Fig. 6*A*; the higher retention time for *E. coli*, *SI Appendix*, Tables S3 and S4).

Intriguingly, all species appeared to have difficulty in passing the middle, 10 µm–wide channels (Fig. 6*C*). All species made U-turns or carried out repeated deflection at different angles, as well as being trapped (*SI Appendix*, Fig. S18). Intuitively, the overall bacterial velocity in meandered channels is the lowest when compared with those in plazas and straight channels (*SI Appendix*, Fig. S19). However, perhaps counterintuitively, the larger-than–5 µm distance between the walls made the trapping effect of the 90°-angled corners more effective, to a near-total extent for *V. natriegens* and *E. coli* and to a lower relative extent for *V. fischeri*, *M. marinus*, and *P. putida*. As expected, the highest passage “success rate” was provided by the meandered channels with the largest distance between walls (Fig. 6*C*). Again, *M. marinus* and *P. putida* executed more zigzagged trajectories than *V. natriegens*, *V. fischeri*, and *E. coli* (Fig. 6*B*).

This complex and species-specific behavior can be explained in view of previous findings as follows. In channels with large gaps between comb teeth, all bacteria can negotiate the passage, their movement being driven mostly by hydrodynamics, with only occasional interference of the local steric interactions between the flagella and the walls. Conversely, in channels with tight confinement, bacteria are also capable of successfully negotiating the channels, this time “channeled” by the local steric interaction between flagella and the 90°-angled walls. Finally, in the channels in the mesoscale region (i.e., 10 µm distance between the “comb teeth”), the mechanism based on hydrodynamics and that based on the local steric interaction do not operate synergistically, increasing the chaotic character of motility and making the overall forward advancement difficult. This is particularly obvious for *V. natriegens* and *E. coli*, which swim parallel to walls (Fig. 3) at a ∼2 µm distance from the walls (Fig. 2*B*), thus leading to frequent U-turns. Additionally, these two species are those with the lowest ratios of the cell body and of the flagella (Fig. 3 and *SI Appendix*, Table S2). The more compact architectures of *V. natriegens* and *E. coli* could explain the near-perfect trapping by frequent circular movements in very confined spaces leading to long retention times. Conversely, but for different reasons, *V. fischeri* (a species swimming the closest to the walls, Fig. 2*B*) and *P. putida* (a species with opportunistic distribution in free volumes) can avoid, to a larger extent than *V. natriegens* and *E. coli*, being trapped in the meandered channels. Tellingly, these two species are also those with the highest ratios of the cell body and of the whole bacterium (Fig. 3 and *SI Appendix*, Table S2). This dichotomy of behavior for species swimming parallel to the walls suggests that the steric interactions–driven movement in tight confinement is also modulated by bacterial shape and not only by size (presented schematically in Fig. 6*D*). Indeed, *V. natriegens* and *E. coli* are both very effectively trapped in mesoscale-sized meandered channels, and while *P. putida*, a much shorter species (*SI Appendix*, Table S2), appeared to have some success, *V. fischeri*, the largest of the species swimming parallel to the walls, had the best success rate. Finally, *M. marinus* was also found to exit mesoscale meandered channels more, but its frequent collision-and-rebound on the walls led to slightly lower trapping efficiencies.

To elucidate whether the trapping effect is permanent or transient, the average duration for successfully traversing the meandered channels was quantified (*SI Appendix*, Table S3). Within the experimental time window (4 to 5 min), *V. natriegens* and *E. coli* were unable to successfully traverse the middle-meandered channels. Although *M. marinus* had a shorter retention time due to its high velocity, the distance that it needed to travel in order to be able to exit the meandered channel was longer. Representative 3D trajectories in meandered channels are presented in *SI Appendix*, Fig. S17. The color-coded trajectories for U-turns, successful passages, and trapped bacteria are presented in *SI Appendix*, Fig. S18.

To conclude, in complex geometries, such as meandered channels, hydrodynamics-driven motility is prevalent in wider channels, and the local steric interactions–based mechanism governs bacterial motility in narrow channels. However, in the mesoscale region, these two mechanisms do not act in synergy, resulting in trapping bacteria, with high efficiency for species swimming parallel to the walls, finely modulated by their characteristic shape ratios.

### Perspectives and Future Work.

The present study, in which we studied a wide range of bacterial motility behavior, provides insights in several areas of applications, as well as suggesting further research.

#### Fundamentals of bacterial motility mechanics in microenvironments.

It was previously shown that a fundamental understanding of the mechanics of the movement of monoflagellated (32, 33, 39) and even biflagellated (61) bacteria in simple geometries, such as the proximity to a surface, can accurately predict motility patterns of bacteria. However, the current study, which described motility patterns of more complex bacterial architectures and in more complex geometries, revealed the limits of this understanding, which would be critical for designing microdevices manipulating bacteria for biosensing, drug delivery, cell sorting, or biocomputation. Further theoretical directions suggested by our study, perhaps coupled with long-term monitoring (74), include analyzing the impact of population variation on cell behavior, investigating the extent to which more complicated bacterial geometries and flagellar arrangements can be represented by more advanced mechanical models, and the need to conduct systematic validation studies. Studies of this type, using artificial microfluidics systems mimicking their natural counterparts, recently carried out for bacteria (75) and fungi (76) or for specifically investigating stochastic processes in bacteria (77), are motivated by the abundance of microbial habitats comprising linear and meandered channels and spaces with different angled turns (*SI Appendix*, Fig. S20).

#### Motility of magnetic bacteria in biological networks.

Chemically or magnetically guided self-propelled bacteria were used for nonsystemic delivery of drugs and cargoes in tumor therapy (78–81). The targeted physiological regions (e.g., deep enteric tissues, hypoxic tumors, tissue granules, and arterioles) (78, 82) are essentially impenetrable to probing devices, but they can be accessed, in principle, by robust bacteria operating as autonomous microrobots moving in the natural microfluidic vascular system (78). The description of bacterial motility, in particular that of *M. marinus*, in PDMS microfluidic channels mimicking the microvascular system surrounding the tumor (e.g., micrometer-range sizes and relevant mechanical elasticity) can lead to the optimization of the operation of these microrobots outside clinical settings, which are expensive to operate and unable to provide reproducible observations at the microscale and in real time.

#### Bacterial cell sorting.

The efficient characterization, sorting, or selection of individual bacterial cells in small volumes are achieved in various microfluidics-based applications, such as those derived from the classical flow cytometry (83–85) to the more recent single-cell analysis (70). In fact, microfluidic devices have been increasingly used for assessing bacterial chemotaxis (86–89), motility (29, 30, 90, 91), and for bacterial cell sorting (46, 72, 91–93), and our results can offer insights for the design of these devices. For instance, the characterization of bacteria as wall accumulators, or wall escapers, can suggest entirely different geometries for microfluidic structures for bacterial cell sorting. Similarly, microfluidic channels can be designed so as to increase retention time (e.g., by having helical profiles) or to amplify the differences in mechanical responses to flow in microfluidics-based flow cytometry.

#### Network-based biocomputation.

Microfluidics-based approaches to computation of problems intractable to electronic computers have been proposed for clique problem (94) and subset sum problem (95). These biological computers require the independent exploration of microfluidic networks encoding a mathematical problem by autonomous agents such as beads (94), cytoskeletal filaments (95), or microorganisms (96). The precision of the microfluidics-based computation is determined by the capacity of biological agents, such as bacteria, to faithfully follow the movement rules embedded in the logic junctions they visit (97). Consequently, the selection of bacterial candidates and the designs of computational microfluidic networks will require the removal or at least minimization of errors, such as U-turns in narrow channels, as well as optimization of the angles of logic gates channels.

## Conclusion

We here provided a comprehensive account of the motility of individual bacterial cells, belonging to five species with considerably varied dimensions and morphologies, in microfluidic networks and with various levels of confinement and complexity. For lesser-confining geometries, such as facing one limiting wall, the motility behavior of the five species studied can be assimilated, with qualifications, to that of monotrichous bacteria with similar dimensions. However, when increasing confinement complexity, as for instance in straight channels with various widths, in networks with exits at various angles, and meandered channels, the classification as swimming parallel to the walls for *V. natriegens*, *E. coli*, *V. fischeri*, and *P. putida* and as escapers, partially, for *E. coli*, *P. putida*, and *M. marinus* is increasingly inaccurate, as a result of the increase of the impact of local steric interaction of species-specific morphology with the tightly confining geometry. The study can be also used as a methodological template for the optimization of the design of microfluidic devices with specific functions (e.g., motility-based cell selection for single-cell genomic screening, detection of rare cells, bacterial entrapment devices for diagnostics, or biocomputation).

## Materials and Methods

All experimental, modeling, and simulation data analysis protocols are presented in *SI Appendix*.

## Supplementary Material

Supplementary File

Supplementary File

Supplementary File

Supplementary File

Supplementary File

## Data Availability

All study data are included in the article and/or supporting information.

## References

[1] A. A. Salyers, D. D. Whitt, Bacterial Pathogenesis: A Molecular Approach (ASM Press, Washington, DC, 1994), vol. 3.

[2] N. Woodford, D. M. Livermore, Infections caused by gram-positive bacteria: A review of the global challenge. J. Infect. 59 (suppl. 1), S4–S16 (2009). 19766888 10.1016/S0163-4453(09)60003-7

[3] P. P. Nagarkar, S. D. Ravetkar, M. G. Watve, Oligophilic bacteria as tools to monitor aseptic pharmaceutical production units. Appl. Environ. Microbiol. 67, 1371–1374 (2001). 11229934 10.1128/AEM.67.3.1371-1374.2001 PMC92737

[4] M. G. Gareau, P. M. Sherman, W. A. Walker, Probiotics and the gut microbiota in intestinal health and disease. Nat. Rev. Gastroenterol. Hepatol. 7, 503–514 (2010). 20664519 10.1038/nrgastro.2010.117 PMC4748966

[5] T. A. Harper., Bioaerosol sampling for airborne bacteria in a small animal veterinary teaching hospital. Infect. Ecol. Epidemiol. 3 (2013). 10.3402/iee.v3i0.20376 PMC3737439 23930156

[6] S. L. Kandel, N. Herschberger, S. H. Kim, S. L. Doty, Diazotrophic endophytes of poplar and willow for growth promotion of rice plants in nitrogen-limited conditions. Crop Sci. 55, 1765–1772 (2015).

[7] Y. Asada, J. Miyake, Photobiological hydrogen production. J. Biosci. Bioeng. 88, 1–6 (1999). 16232564 10.1016/s1389-1723(99)80166-2

[8] A. Esmaeili, A. A. Pourbabaee, H. A. Alikhani, F. Shabani, E. Esmaeili, Biodegradation of low-density polyethylene (LDPE) by mixed culture of Lysinibacillus xylanilyticus and Aspergillus Niger in soil. PLoS One 8, e71720 (2013). 24086254 10.1371/journal.pone.0071720 PMC3781136

[9] M. Höckenreiner, H. Neugebauer, L. Elango, Ex situ bioremediation method for the treatment of groundwater contaminated with PAHs. Int. J. Environ. Sci. Technol. 12, 285–296 (2015).

[10] F. Reith, C. M. Zammit, S. L. Rogers, D. C. McPhail, J. Brugger, Potential utilisation of micro-organisms in gold processing: A review. Miner. Process. Extr. Metall. 121, 251–260 (2012).

[11] O. Habimana, A. Semião, E. Casey, The role of cell-surface interactions in bacterial initial adhesion and consequent biofilm formation on nanofiltration/reverse osmosis membranes. J. Membrane Sci. 454, 82–96 (2014).

[12] C. Verde, D. Giordano, C. M. Bellas, G. di Prisco, A. M. Anesio, “Polar marine microorganisms and climate change” in Advances in Microbial Physiology, R. K. Poole, Ed. (Elsevier, 2016), 69, pp. 187–215. 10.1016/bs.ampbs.2016.07.002 27720011

[13] G. H. Wadhams, J. P. Armitage, Making sense of it all: Bacterial chemotaxis. Nat. Rev. Mol. Cell Biol. 5, 1024–1037 (2004). 15573139 10.1038/nrm1524

[14] A. Z. Komaromy., Arrays of nano-structured surfaces to probe the adhesion and viability of bacteria. Microelectron. Eng. 91, 39–43 (2012).

[15] A. Persat., The mechanical world of bacteria. Cell 161, 988–997 (2015). 26000479 10.1016/j.cell.2015.05.005 PMC4451180

[16] O. Felfoul, S. Martel, Assessment of navigation control strategy for magnetotactic bacteria in microchannel: Toward targeting solid tumors. Biomed. Microdevices 15, 1015–1024 (2013). 23857666 10.1007/s10544-013-9794-4

[17] K. F. Jarrell, M. J. McBride, The surprisingly diverse ways that prokaryotes move. Nat. Rev. Microbiol. 6, 466–476 (2008). 18461074 10.1038/nrmicro1900

[18] M. E. J. Holwill, R. E. Burge, A hydrodynamic study of the motility of flagellated bacteria. Arch. Biochem. Biophys. 101, 249–260 (1963). 13961491 10.1016/s0003-9861(63)80010-7

[19] F. Bai., Conformational spread as a mechanism for cooperativity in the bacterial flagellar switch. Science 327, 685–689 (2010). 20133571 10.1126/science.1182105

[20] F. F. Chevance, K. T. Hughes, Coordinating assembly of a bacterial macromolecular machine. Nat. Rev. Microbiol. 6, 455–465 (2008). 18483484 10.1038/nrmicro1887 PMC5963726

[21] K. D. Young, Bacterial morphology: Why have different shapes? Curr. Opin. Microbiol. 10, 596–600 (2007). 17981076 10.1016/j.mib.2007.09.009 PMC2169503

[22] A. Bren, M. Eisenbach, How signals are heard during bacterial chemotaxis: Protein-protein interactions in sensory signal propagation. J. Bacteriol. 182, 6865–6873 (2000). 11092844 10.1128/jb.182.24.6865-6873.2000 PMC94809

[23] R. M. Harshey, Bacterial motility on a surface: Many ways to a common goal. Annu. Rev. Microbiol. 57, 249–273 (2003). 14527279 10.1146/annurev.micro.57.030502.091014

[24] J. G. Mitchell, K. Kogure, Bacterial motility: Links to the environment and a driving force for microbial physics. FEMS Microbiol. Ecol. 55, 3–16 (2006). 16420610 10.1111/j.1574-6941.2005.00003.x

[25] P. Denissenko, V. Kantsler, D. J. Smith, J. Kirkman-Brown, Human spermatozoa migration in microchannels reveals boundary-following navigation. Proc. Natl. Acad. Sci. U.S.A. 109, 8007–8010 (2012). 22566658 10.1073/pnas.1202934109 PMC3361448

[26] K. Drescher, J. Dunkel, L. H. Cisneros, S. Ganguly, R. E. Goldstein, Fluid dynamics and noise in bacterial cell-cell and cell-surface scattering. Proc. Natl. Acad. Sci. U.S.A. 108, 10940–10945 (2011). 21690349 10.1073/pnas.1019079108 PMC3131322

[27] L. Lemelle, J. F. Palierne, E. Chatre, C. Place, Counterclockwise circular motion of bacteria swimming at the air-liquid interface. J. Bacteriol. 192, 6307–6308 (2010). 20889751 10.1128/JB.00397-10 PMC2981220

[28] H. C. Berg, L. Turner, Chemotaxis of bacteria in glass capillary arrays. Escherichia coli, motility, microchannel plate, and light scattering. Biophys. J. 58, 919–930 (1990). 2248995 10.1016/S0006-3495(90)82436-X PMC1281037

[29] M. Binz, A. P. Lee, C. Edwards, D. V. Nicolau, Motility of bacteria in microfluidic structures. Microelectron. Eng. 87, 810–813 (2010).

[30] B. Libberton, M. Binz, H. van Zalinge, D. V. Nicolau, Efficiency of the flagellar propulsion of Escherichia coli in confined microfluidic geometries. Phys. Rev. E 99, 012408 (2019). 30780339 10.1103/PhysRevE.99.012408

[31] D. Giacché, T. Ishikawa, T. Yamaguchi, Hydrodynamic entrapment of bacteria swimming near a solid surface. Phys. Rev. E Stat. Nonlin. Soft Matter Phys. 82, 056309 (2010). 21230578 10.1103/PhysRevE.82.056309

[32] H. Shum, E. A. Gaffney, D. J. Smith, Modelling bacterial behaviour close to a no-slip plane boundary: The influence of bacterial geometry. Proc. Royal Soc. Math. Phys. Eng. Sci. 466, 1725–1748 (2010).

[33] H. Shum, E. A. Gaffney, The effects of flagellar hook compliance on motility of monotrichous bacteria: A modeling study. Phys. Fluids 24, 061901 (2012).

[34] A. Acemoglu, S. Yesilyurt, Effects of geometric parameters on swimming of micro organisms with single helical flagellum in circular channels. Biophys. J. 106, 1537–1547 (2014). 24703315 10.1016/j.bpj.2014.01.047 PMC3976525

[35] H. Shum, E. A. Gaffney, Hydrodynamic analysis of flagellated bacteria swimming near one and between two no-slip plane boundaries. Phys. Rev. E Stat. Nonlin. Soft Matter Phys. 91, 033012 (2015). 25871207 10.1103/PhysRevE.91.033012

[36] J. Hu, A. Wysocki, R. G. Winkler, G. Gompper, Physical sensing of surface properties by microswimmers–Directing bacterial motion via wall slip. Sci. Rep. 5, 9586 (2015). 25993019 10.1038/srep09586 PMC4438609

[37] Y. Park, Y. Kim, S. Lim, Flagellated bacteria swim in circles near a rigid wall. Phys. Rev. E 100, 063112 (2019). 31962483 10.1103/PhysRevE.100.063112

[38] E. Lauga, Bacterial hydrodynamics. Annu. Rev. Fluid Mech. 48, 105–130 (2016).

[39] H. Shum, E. A. Gaffney, Hydrodynamic analysis of flagellated bacteria swimming in corners of rectangular channels. Phys. Rev. E Stat. Nonlin. Soft Matter Phys. 92, 063016 (2015). 26764813 10.1103/PhysRevE.92.063016

[40] E. K. Sackmann, A. L. Fulton, D. J. Beebe, The present and future role of microfluidics in biomedical research. Nature 507, 181–189 (2014). 24622198 10.1038/nature13118

[41] J. Zhang., Fundamentals and applications of inertial microfluidics: A review. Lab Chip 16, 10–34 (2016). 26584257 10.1039/c5lc01159k

[42] D. Erickson, D. Q. Li, Integrated microfluidic devices. Anal. Chim. Acta 507, 11–26 (2004).

[43] G.-X. Zheng., An integrated microfludic device for culturing and screening of Giardia lamblia. Exp. Parasitol. 137, 1–7 (2014). 24316463 10.1016/j.exppara.2013.11.009

[44] C.-X. Xu, X.-F. Yin, Continuous cell introduction and rapid dynamic lysis for high-throughput single-cell analysis on microfludic chips with hydrodynamic focusing. J. Chromatogr. A 1218, 726–732 (2011). 21185567 10.1016/j.chroma.2010.11.049

[45] D. Yuan., Sheathless separation of microalgae from bacteria using a simple straight channel based on viscoelastic microfluidics. Lab Chip 19, 2811–2821 (2019). 31312819 10.1039/c9lc00482c

[46] L. Y. Yeo, H. C. Chang, P. P. Chan, J. R. Friend, Microfluidic devices for bioapplications. Small 7, 12–48 (2011). 21072867 10.1002/smll.201000946

[47] T. Kalisky, S. R. Quake, Single-cell genomics. Nat. Methods 8, 311–314 (2011). 21451520 10.1038/nmeth0411-311

[48] R. U. Sheth, S. S. Yim, F. L. Wu, H. H. Wang, Multiplex recording of cellular events over time on CRISPR biological tape. Science 358, 1457–1461 (2017). 29170279 10.1126/science.aao0958 PMC7869111

[49] B. J. Kim, M. Wu, Microfluidics for mammalian cell chemotaxis. Ann. Biomed. Eng. 40, 1316–1327 (2012). 22189490 10.1007/s10439-011-0489-9 PMC3424276

[50] K. L. Hanson., Fungi use efficient algorithms for the exploration of microfluidic networks. Small 2, 1212–1220 (2006). 17193591 10.1002/smll.200600105

[51] M. Held, A. P. Lee, C. Edwards, D. V. Nicolau, Microfluidics structures for probing the dynamic behaviour of filamentous fungi. Microelectron. Eng. 87, 786–789 (2010).

[52] J. Wang., Detection of size spectrum of microalgae cells in an integrated underwater microfluidic device. J. Exp. Mar. Biol. Ecol. 473, 129–137 (2015).

[53] Z. Liu, K. D. Papadopoulos, Unidirectional motility of Escherichia coli in restrictive capillaries. Appl. Environ. Microbiol. 61, 3567–3572 (1995). 7486991 10.1128/aem.61.10.3567-3572.1995 PMC167651

[54] G. S. Kijanka, I. K. Dimov, R. Burger, J. Ducrée, Real-time monitoring of cell migration, phagocytosis and cell surface receptor dynamics using a novel, live-cell opto-microfluidic technique. Anal. Chim. Acta 872, 95–99 (2015). 25892074 10.1016/j.aca.2014.12.035

[55] M. Nayak, A. S. Perumal, D. V. Nicolau, F. C. M. J. M. Van Delft, Bacterial motility behaviour in sub-ten micron wide geometries” in *2018 16th IEEE International New Circuits and Systems Conference*, R. Izquierdo, A. Miled, Eds. NEWCAS 2018 (Montreal, QC, 2018), pp. 382–384.

[56] A. S. Perumal, M. Nayak, V. Tokárová, O. Kašpar, D. V. Nicolau, “Space partitioning and maze solving by bacteria” in *Proceedings of the lecture Notes of the Institute for Computer Sciences, Social-Informatics and Telecommunications Engineering, LNICST*, A. Compagnoni, W. Casey, Y. Cai, B. Mishra, Eds. (Pittsburgh, PA, 2019), pp. 175–180.

[57] A. P. Berke, L. Turner, H. C. Berg, E. Lauga, Hydrodynamic attraction of swimming microorganisms by surfaces. Phys. Rev. Lett. 101, 038102 (2008). 18764299 10.1103/PhysRevLett.101.038102

[58] E. Lauga, W. R. DiLuzio, G. M. Whitesides, H. A. Stone, Swimming in circles: Motion of bacteria near solid boundaries. Biophys. J. 90, 400–412 (2006). 16239332 10.1529/biophysj.105.069401 PMC1367047

[59] E. P. Ipina, S. Otte, R. Pontier-Bres, D. Czerucka, F. Peruani, Bacteria display optimal transport near surfaces. Nat. Phys. 15, 610–615 (2019).

[60] A. S. Utada., Vibrio cholerae use pili and flagella synergistically to effect motility switching and conditional surface attachment. Nat. Commun. 5, 4913 (2014). 25234699 10.1038/ncomms5913 PMC4420032

[61] H. Shum, Microswimmer propulsion by two steadily rotating helical flagella. Micromachines (Basel) 10, 65 (2019). 10.3390/mi10010065 PMC6356978 30669288

[62] K. Bente., High-speed motility originates from cooperatively pushing and pulling flagella bundles in bilophotrichous bacteria. eLife 9, e47551 (2020). 31989923 10.7554/eLife.47551 PMC7010408

[63] W. R. DiLuzio., Escherichia coli swim on the right-hand side. Nature 435, 1271–1274 (2005). 15988531 10.1038/nature03660

[64] M. Theves, J. Taktikos, V. Zaburdaev, H. Stark, C. Beta, Random walk patterns of a soil bacterium in open and confined environments. EPL 109, 28007 (2015).

[65] H. Shum, “Simulations and modelling of bacterial flagellar propulsion,” PhD thesis, University of Oxford, Oxford, UK (2011).

[66] S. Bianchi, F. Saglimbeni, R. Di Leonardo, Holographic imaging reveals the mechanism of wall entrapment in swimming bacteria. Phys. Rev. X 7, 011010 (2017).

[67] P. D. Frymier, R. M. Ford, H. C. Berg, P. T. Cummings, Three-dimensional tracking of motile bacteria near a solid planar surface. Proc. Natl. Acad. Sci. U.S.A. 92, 6195–6199 (1995). 7597100 10.1073/pnas.92.13.6195 PMC41669

[68] N. Figueroa-Morales., E. coli “super-contaminates” narrow ducts fostered by broad run-time distribution. Sci. Adv. 6, eaay0155 (2020). 32201716 10.1126/sciadv.aay0155 PMC7069694

[69] J. Männik, R. Driessen, P. Galajda, J. E. Keymer, C. Dekker, Bacterial growth and motility in sub-micron constrictions. Proc. Natl. Acad. Sci. U.S.A. 106, 14861–14866 (2009). 19706420 10.1073/pnas.0907542106 PMC2729279

[70] P. C. Blainey, The future is now: Single-cell genomics of bacteria and archaea. FEMS Microbiol. Rev. 37, 407–427 (2013). 23298390 10.1111/1574-6976.12015 PMC3878092

[71] B. M. Paegel, G. F. Joyce, Microfluidic compartmentalized directed evolution. Chem. Biol. 17, 717–724 (2010). 20659684 10.1016/j.chembiol.2010.05.021 PMC2912841

[72] P. Galajda, J. Keymer, P. Chaikin, R. Austin, A wall of funnels concentrates swimming bacteria. J. Bacteriol. 189, 8704–8707 (2007). 17890308 10.1128/JB.01033-07 PMC2168927

[73] T. V. Phan. Bacterial route finding and collective escape in mazes and fractals. Phys. Rev. X 10, 031017 (2020).

[74] F. K. Balagaddé, L. You, C. L. Hansen, F. H. Arnold, S. R. Quake, Long-term monitoring of bacteria undergoing programmed population control in a microchemostat. Science 309, 137–140 (2005). 15994559 10.1126/science.1109173

[75] H. Massalha, E. Korenblum, S. Malitsky, O. H. Shapiro, A. Aharoni, Live imaging of root-bacteria interactions in a microfluidics setup. Proc. Natl. Acad. Sci. U.S.A. 114, 4549–4554 (2017). 28348235 10.1073/pnas.1618584114 PMC5410799

[76] M. Held, O. Kašpar, C. Edwards, D. V. Nicolau, Intracellular mechanisms of fungal space searching in microenvironments. Proc. Natl. Acad. Sci. U.S.A. 116, 13543–13552 (2019). 31213536 10.1073/pnas.1816423116 PMC6613077

[77] L. Potvin-Trottier, S. Luro, J. Paulsson, Microfluidics and single-cell microscopy to study stochastic processes in bacteria. Curr. Opin. Microbiol. 43, 186–192 (2018). 29494845 10.1016/j.mib.2017.12.004 PMC6044433

[78] S. Martel, Swimming microorganisms acting as nanorobots versus artificial nanorobotic agents: A perspective view from an historical retrospective on the future of medical nanorobotics in the largest known three-dimensional biomicrofluidic networks. Biomicrofluidics 10, 021301 (2016). 27158285 10.1063/1.4945734 PMC4841799

[79] S. Martel, C. C. Tremblay, S. Ngakeng, G. Langlois, Controlled manipulation and actuation of micro-objects with magnetotactic bacteria. Appl. Phys. Lett. 89, 233904(2006).

[80] H. Terashima, S. Kojima, M. Homma, Flagellar motility in bacteria structure and function of flagellar motor. Int. Rev. Cell Mol. Biol. 270, 39–85 (2008). 19081534 10.1016/S1937-6448(08)01402-0

[81] D. Akin., Bacteria-mediated delivery of nanoparticles and cargo into cells. Nat. Nanotechnol. 2, 441–449 (2007). 18654330 10.1038/nnano.2007.149

[82] S. Martel, Bacterial microsystems and microrobots. Biomed. Microdevices 14, 1033–1045 (2012). 22960952 10.1007/s10544-012-9696-x

[83] B. P. Tracy, S. M. Gaida, E. T. Papoutsakis, Flow cytometry for bacteria: Enabling metabolic engineering, synthetic biology and the elucidation of complex phenotypes. Curr. Opin. Biotechnol. 21, 85–99 (2010). 20206495 10.1016/j.copbio.2010.02.006

[84] D. Huh, W. Gu, Y. Kamotani, J. B. Grotberg, S. Takayama, Microfluidics for flow cytometric analysis of cells and particles. Physiol. Meas. 26, R73–R98 (2005). 15798290 10.1088/0967-3334/26/3/R02

[85] J. Oakey., Particle focusing in staged inertial microfluidic devices for flow cytometry. Anal. Chem. 82, 3862–3867 (2010). 20373755 10.1021/ac100387b PMC3136802

[86] X. Wang, J. Atencia, R. M. Ford, Quantitative analysis of chemotaxis towards toluene by Pseudomonas putida in a convection-free microfluidic device. Biotechnol. Bioeng. 112, 896–904 (2015). 25408100 10.1002/bit.25497

[87] J. A. Crooks, M. D. Stilwell, P. M. Oliver, Z. Zhong, D. B. Weibel, Decoding the chemical language of motile bacteria by using high-throughput microfluidic assays. ChemBioChem 16, 2151–2155 (2015). 26285783 10.1002/cbic.201500324 PMC4665984

[88] H. H. Jeong., Microfluidic monitoring of Pseudomonas aeruginosa chemotaxis under the continuous chemical gradient. Biosens. Bioelectron. 26, 351–356 (2010). 20810268 10.1016/j.bios.2010.08.006

[89] H. Kim, J. Ali, K. Phuyal, S. Park, M. J. Kim, Investigation of bacterial chemotaxis using a simple three-point microfluidic system. Biochip J. 9, 50–58 (2015).

[90] O. Sipos, K. Nagy, P. Galajda, Patterns of collective bacterial motion in microfluidic devices. Chem. Biochem. Eng. Q. 28, 233–240 (2014).

[91] B. Kaehr, J. B. Shear, High-throughput design of microfluidics based on directed bacterial motility. Lab Chip 9, 2632–2637 (2009). 19704977 10.1039/b908119d

[92] S. Park, D. Kim, R. J. Mitchell, T. Kim, A microfluidic concentrator array for quantitative predation assays of predatory microbes. Lab Chip 11, 2916–2923 (2011). 21761042 10.1039/c1lc20230h

[93] Z. Wu, B. Willing, J. Bjerketorp, J. K. Jansson, K. Hjort, Soft inertial microfluidics for high throughput separation of bacteria from human blood cells. Lab Chip 9, 1193–1199 (2009). 19370236 10.1039/b817611f

[94] D. T. Chiu, E. Pezzoli, H. Wu, A. D. Stroock, G. M. Whitesides, Using three-dimensional microfluidic networks for solving computationally hard problems. Proc. Natl. Acad. Sci. U.S.A. 98, 2961–2966 (2001). 11248014 10.1073/pnas.061014198 PMC30589

[95] D. V. Nicolau Jr., Parallel computation with molecular-motor-propelled agents in nanofabricated networks. Proc. Natl. Acad. Sci. U.S.A. 113, 2591–2596 (2016). 26903637 10.1073/pnas.1510825113 PMC4791004

[96] D. V. Nicolau., Molecular motors-based micro- and nano-biocomputation devices. Microelectron. Eng. 83, 1582–1588 (2006).

[97] F. C. M. J. M. van Delft., Something has to give: Scaling combinatorial computing by biological agents exploring physical networks encoding NP-complete problems. Interface Focus 8, 20180034 (2018). 30443332 10.1098/rsfs.2018.0034 PMC6227808

