# Supplementary material for: Patterns of bacterial motility in microfluidics-confining environments
Source: Proc Natl Acad Sci U S A. 2021 Apr 19;118(17):e2013925118. doi: 10.1073/pnas.2013925118 (PMC8092623; doi:10.1073/pnas.2013925118)
Supplement: Supplementary File [file pnas.2013925118.sapp.pdf]

SUPPORTING INFORMATION

Patterns of bacterial motility in microfluidics-confining environments

*Viola Tokárová, Ayyappasamy Sudalaiyadum Perumal, Monalisha Nayak, Henry Shum, Ondřej Kašpar, Kavya Rajendran, Mahmood Mohammadi, Charles Tremblay, Eamonn Andrew Gaffney, Sylvain Martel, Dan V. Nicolau Jr., Dan V. Nicolau**

1. Detailed Experimental Section

1.1. Experimental protocols

1.1.1. *Bacterial species*

Five bacterial species living in micro-environments were studied, presented here in an increased order of architectural complexity. *Vibrio natriegens* is a rod-shaped, polar uni-flagellated bacterium.(1, 2) *Magnetococcus marinus* (MC-1) is a spherical bacterium with two clusters of seven flagella at one polar end (3, 4), *Pseudomonas putida* (ATCC® 12633™) (5, 6) and *Vibrio fischeri* (Ward's Science 15-5722) have rod-shaped bodies,(7, 8) and are polar-multi-flagellated bacteria. *Escherichia coli* MG1665 (K12-wild type) is a rod-shaped bacterium with a peritrichous flagellar machinery.(9-12) **Figure 1** (main text) and **Table S1** present the characteristics of the five bacterial strains used in this study, and **Figure S1** presents detailed SEM images of individual bacterial cells.

All the cultures, except *M. marinus*, were maintained in agar plates and cultured in Luria-Bertani (LB) medium prior to the experiments. *P. putida* and *V. fischeri* were cultivated at room temperature (RT), as reported elsewhere (5) while *E. coli* and *V. natriegens* were cultivated at 30ºC. *E. coli* and *V. natriegens* were genetically transformed with a plasmid to express mCherry for visualization and tracking in microfluidic devices. *E. coli* and *V. natriegens* were transformed to constitutively express the plasmid pMF440-mChe (a gift from Dr. Michael Franklin’s lab, Addgene Plasmid #62550). The plasmids express mCherry, a red fluorescent protein in bacteria to visualize them in our experiments using fluorescence microscopy techniques. *M. marinus* was cultivated and maintained at École Polytechnique de Montréal, Canada, in a microaerophilic, chemo-hetero lithotrophic chemically-defined medium, rich with ferrous ions, grown in dark, at room temperature, as described earlier (3), while a 24 hrs to 48 hrs culture was used for microfluidic experiments.

1.1.2. *Design and fabrication of the microfluidics networks*

The microfluidic chip for probing bacterial motility (**Figure 1C**, first left) comprises parallel reservoirs with widths of 2 mm, connected through 1 mm-wide ‘bus’ channel (**Figure 1C**, second left). Every area consists of 1 mm x 1 mm structures of 5 x 5 identical geometries separated by open spaces (‘plazas’) of 100 µm x 100 µm (**Figure 1C**, third left).

The quantification of specific motility parameters required specific designs of the microfluidic structures **(Figure 1C**, third left, clockwise direction), as follows: (i) a set of linear, 100 µm-long channels (**Figure S2A**) with various widths, i.e., 2, 3, 4, 5, 6, 7 and 8 µm, probed the linear movement and possible U-turns (~ 180°); (ii) zig-zag channels, ‘combs’, with 5, 10, and 15 µm-long teeth length (**Figure S2C**), presenting 90° angles for each tooth, probed the corner preference and wall guiding behavior; (iii) 3.5 µm-wide channels (**Figure S2B**) presenting different sideways angles, i.e., 0°, 30°, 45°, 60°, 90°, 120°,

135°, and 150°, probed the deflection of movement and turn angle preferences; and (iv) 100 x 100 µm chambers, 'plazas', with two entrances opposite to each other on opposite walls (**Figure S2**).

The microfluidic chip was made of polydimethylsiloxane (PDMS) through the replication of a positive-relief silicon master, fabricated by standard photolithography.(9, 13) The mixture of PDMS and cross-linker (weight ratio 10:1) was poured onto the silicon master, degassed inside vacuum chamber to remove air bubbles, and cured at 65°C overnight to ensure full cross-linking. After cutting and peeling off, the PDMS replica was treated in air plasma for 30 seconds to render the surface hydrophilic, before irreversibly bonding it onto the glass coverslip (also plasma-activated for 30 seconds).

1.1.3. *Impact of the distance between horizontal planes*

In chambers with lateral dimensions considerably larger than the size of bacterial cells, and if the horizontal surfaces are placed at a distance that allows the decoupling of their impact, bacterial motility is limited only by one of the horizontal planes (and by the vertical walls and corners placed at large distances from each other). However, to avoid the sagging, or outright collapse of the top horizontal plane in PDMS microfluidic chambers, the optimal height/width ratio is around 0.05,(14) i.e., approximately 5 µm for a 100x100 µm chamber. Because bacteria approach walls diagonally, a conservative design of the microfluidics structures required that the distance between the horizontal walls be larger than the vertical projection of the bacterium length (cell body and flagella) at a 45° diagonal. For the dimensions of the bacteria studied, this condition was fulfilled, for all species, by a height of 6 µm (**Figure 1A, Table S1**). Conversely, this condition was not fulfilled for any of the species studied at a height of 4 µm, with *E. coli, V. fischeri*, *M. marinus*, *P. putida,* and *V. natriegens*, exceeding the 4 µm clearance, in this order (**Figure 1B**).

Indeed, the motility behavior in large chambers with low ceilings presented evidence of the coupling of the impact on both horizontal planes on bacterial motility, i.e., a considerable alteration of the distribution of deflection angles of the 2D projections of bacterial trajectories (**Figures S3**), which appeared to be more pronounced for species with characteristic length larger than the 4 µm clearance. A finer analysis of the bacterial 3D trajectories revealed a much narrower distribution of the curvatures of these trajectories, for all species, for 4 µm tall plazas compared with those in 6 µm tall plazas (**Figure S4**). These streamlined trajectories appear be the result of the increased confinement by both horizontal walls of the chambers with 4 µm heights.

A similar analysis for plazas with 4 µm heights (**Figure S5**) and the characteristic trajectories (presented in **Movie S1**) also showed important differences in the motility behavior of all bacteria. Consequently, more detailed motility experiments were performed extensively in 6 µm-tall microfluidic structures.

1.1.4. *Motility experiments.*

Immediately after sealing the PDMS structure on the coverslip, the microfluidics chip was flooded with the working buffer, i.e., LB medium for *V. natriegens, P. putida*, *V. fischeri*, *E. coli*, and Phosphate-Buffered Saline (PBS) buffer for *M. marinus*, for 1 hour to pre-wet the microfluidic structure, then stored inside a wet chamber at 4°C before use. Separately, a log-phase bacterial suspension was introduced into the microfluidics chamber through the open ends of the PDMS stamps and left in contact for few minutes to allow of bacteria to enter the

channels and plazas (**Figure 1C**, first on the left). To ensure that the bacterial chemotaxis-free motility is the only, or the overriding mechanism at play, the working fluids have an excess of nutrients, and the experimental time is short enough (few minutes inside the confined environment) to ensure that the level of nutrients remains practically constant. Furthermore, the bacterial population in microenvironments never reached the population density of log phase or stationary phase (15, 16), during which other factors, e.g., quorum sensing, or chemotaxis could play a role in space searching and foraging for nutrients.

1.1.5. *Image acquisition and analysis*

Bacterial flagella were stained using Hardy Diagnostics Flagella Stain following the product protocol and visualized by optical microscopy (Olympus IX83, U PLAN S-APO 100X oil objective). The scanning electron microscopy images of bacterial cells and PDMS structures were obtained using a Quanta FEI450 SEM and Hitachi S-3400N SEM system. The image acquisition of *M. marinus* used a specially designed inverted Zeiss AxioImager Z1m microscope with AxioVision Software Sonny HD-1000 camera (with VirtualDub 1.10.4 software), LD Epiplan 20x (NA 0.4) and N-Achroplan 10x (NA 0.25) objectives. The dark field imaging system enhances the contrast between bacterial cell and surrounding structures, which is necessary for further image analysis of fast swimming bacteria such as *M. marinus*. The experiments with *V. natriegens, P. putida, V. fischeri,* and *E. coli* were performed on a system mounted on an inverted, Spinning Disk Confocal Olympus IX83 microscope, with MetaMorph® Microscopy Automation & Image Analysis Software (Molecular Devices), and 10x (NA 0.4), 20x (NA 0.75) and 40x (NA 0.95) Differential Interference Contrast (DIC) objectives. The duration of image acquisition was based on type of imaging like bright field microscopy (for *M. marinus* MC-1 and *P. putida*) or with fluorescence microscopy (*E. coli, V. natriegens*, *V. fischeri*). Depending on the type of acquisition, a different exposure was used, and the frames used for plotting density maps or another trajectory analysis were normalized accordingly. Density maps also used the average number of bacteria per frame.

ImageJ 1.50a (17), a public domain software, has been used for image analysis, density map reconstructions, and time-resolved bacterial tracking. The density maps of bacterial movement inside confined structures were prepared from original RGB image stack as follows: (i) a median of 8-bit image stack (a background) was created using a ‘Z Project’ function; (ii) a new stack was created as a difference between the original stack and the background; (iii) the histogram of a new stack was adjusted and converted to a binary image; (iv) the binary operations ‘Close’ and ‘Dilate’ were applied to remove the remaining noise; and (v) all binary images were superimposed into one image.

In some cases, the density map created by the procedure described above was accompanied by a high level of noise near the walls of the channels, due to the non-uniform light conditions in the optically transparent PDMS chip. In such cases, the original stack of n-images was duplicated in the range of 1 to *n*-1 and 2 to *n*. Thus, the difference |Img2 - Img1| between stacks shifted by one frame highlighted the bacterial movement without the background and light-related noise. This difference, used to calculate the absolute change between two images, is important, since bacteria have also free movement along the vertical z-axis, in and out of the focal plane, in the range limited by the height of the PDMS structures. The new image stack was processed by steps (iii)-(v) described above to create the respective density maps.

The trajectories of the single bacterium were tracked by the automatic (TrackMate, ImageJ), and the manual (MTrackJ, ImageJ) plugins (18). The TrackMate plugin, using LAP tracker

algorithm, was used for *M. marinus*, which exhibits a quasi-linear motion, with low deflection angles (see Results Section 2.1), thus making the analysis amenable to automation. The settings for this 2-parameter tracker were chosen to reach the maximum distance between the two consecutive points of one trajectory, at a given time, with a time gap set to three frames. However, because *P. putida* and *V. fischeri* tend to swim along the walls, thus leading to the possibility of interrupted trajectories, the automatic tracking required more statistical data. The image analysis of these species used the manual MTrackJ plugin, with a dark/white centroid snap feature, with a point-and-click tracking. To facilitate automatic tracking, we used fluorescently labelled *E. coli* and *V. natriegens*. In all cases, the acquired x-y-time coordinates were used for calculating the velocity and the deflection angle from trajectories of individual bacteria. All experiments and image analysis were performed in biological replicates, with at least 3 sets of experiment for each bacterial species in each motility structure. For analysis and probability/fraction % representation, at least 200-300 independent bacterial count were used in multiple sets, to obtain a high confidence data and a statistical significance of $P<0.005$. For the density map, a fixed number of frames with average bacterial count ($n=18\pm7$) on each frame in any particular motility structure for any bacterial species was kept standard, so that the density map intensity was comparable to minimum and maximum values for each motility structure under discussion.

1.1.6. Imaging for mapping 3D motility patterns of bacteria in 6 μm plaza

For imaging bacterial trajectories in 3D, we used the piezo-stage controller of Olympus IX83, confocal microscope for rapid acquisition of z-stacking of the bacterial trajectory. The devices of 6 μm tall plaza were z-stacked at a step size of 2 μm for 12 μm. In total 7 images were captured at an exposure of 10 ms (a total of ~90 ms to 100 ms per time point). For plotting trajectory analysis, the tracking was done manually, followed by the intensity-based segregation of trajectory points for each z-planes with highest fluorescence or bright field intensity.(19) This highest intensity bacterial corresponds to ‘in focus’ bacterial trajectories and marks the z-plane maximum for each time point. We used ‘Origin’ software for plotting the 3D tracks as described in the Results and discussion section.

1.2. Bacterial species

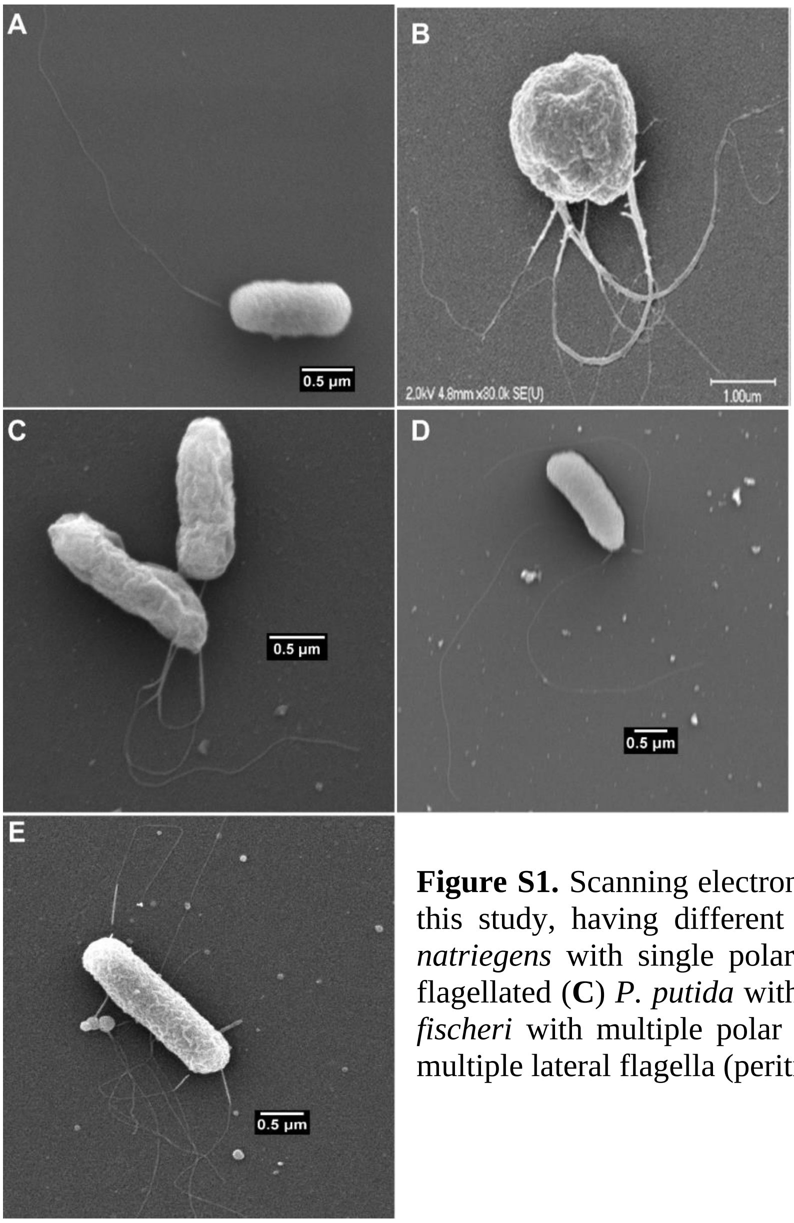

Figure S1. Scanning electron images of the bacteria used in this study, having different flagellar architectures. (**A**) *V. natriegens* with single polar flagella; (**B**) *M. marinus*, bi-flagellated (**C**) *P. putida* with multiple polar flagella; (**D**) *V. fischeri* with multiple polar flagella; (**E**) *E. coli* K12 with multiple lateral flagella (peritrichous arrangement).

Table S1. Characteristics of the motile bacteria used in this study

**Bacteria**	***V. natriegens***	***M. marinus***	***P. putida***	***V. fischeri***	***E. coli***
Cell:<br>Length [µm]<br>Width [µm]	 1.5 ± 0.5<br>0.9 ± 0.1	 1.9 ± 0.2<br>1.9 ± 0.2	 1.6 ± 0.3<br>0.6 ± 0.2	 1.8 ± 0.1<br>0.6 ± 0.1	 1.9 ± 0.6<br>1.0 ± 0.2
Flagellum/-a<br>Architecture<br>Length [µm]	 One<br>4.7 ± 0.9	 Two bundles<br>4.0 ± 0.8	 Polar multiple<br>3.9 ± 0.8	 Polar multiple<br>5.1 ± 1.1	 Peritrichous<br>4.7 ± 1.4
Velocity [µm.s^{-1}]	15-20	Up to 200	27 - 44	60-100	< 20
Habitat	Marine, or fresh water	Marine	Soil	Marine (free; or fish, squid)	Ubiquitous, intestine/gut
Aerobic	Aerobic	Micro-aerophilic	Aerobic	Aerobic	Facultative anaerobic
Media	LB, LB-V2 salt medium	Chemo-hetero-lithotrophic	LB broth/agar	LB broth/agar	LB broth/agar

1.3. Microfluidics structures and their characteristic dimensions

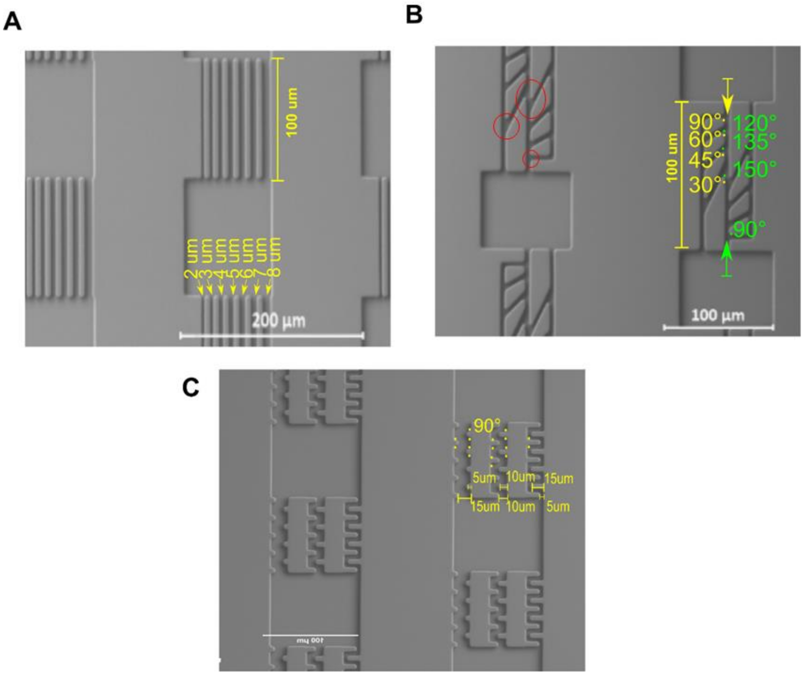

Figure S2. Scanning electron microscopy (SEM) images of PDMS microfluidic structures for the probing of the bacterial motility, separated by quasi-open spaces. **A**. Linear channels with different widths, from 2 µm to 8 µm. **B.** Turn-angle chip presenting eight different angles (0°, or straight path, and 30°, 45°, 60°, 90°, 120°, 135°, and 150° angles). Note the various volumes available for motility at the intersection of the axial and lateral angle, for 90°, 30°/150°, and 150° angles (red circles, top left). **C.** Meandered channels with three different tooth lengths (5 µm, 10 µm, and 15 µm).

2. Results and Discussion

2.1. Motility in large chambers

2.1.1. Impact of the distance between horizontal planes

2.1.1.1. Turn angle preference from 2D trajectories

To assess the possible coupling of the interaction of the horizontal plane in plazas, for 4 and 6 µm heights, respectively, the *turn angle preference* of motility was calculated, in the first instance, using 2D projections of the 3D trajectories (**Figure S3**).

It was found that the turn angle preference was considerably different for plazas with 4 µm, and 6 µm heights, respectively. Importantly, this difference appears to be larger for bacteria whose characteristic lengths are larger than the clearance of 4 µm heights (**Figure 1B**), with the notable exception of *E. coli*.

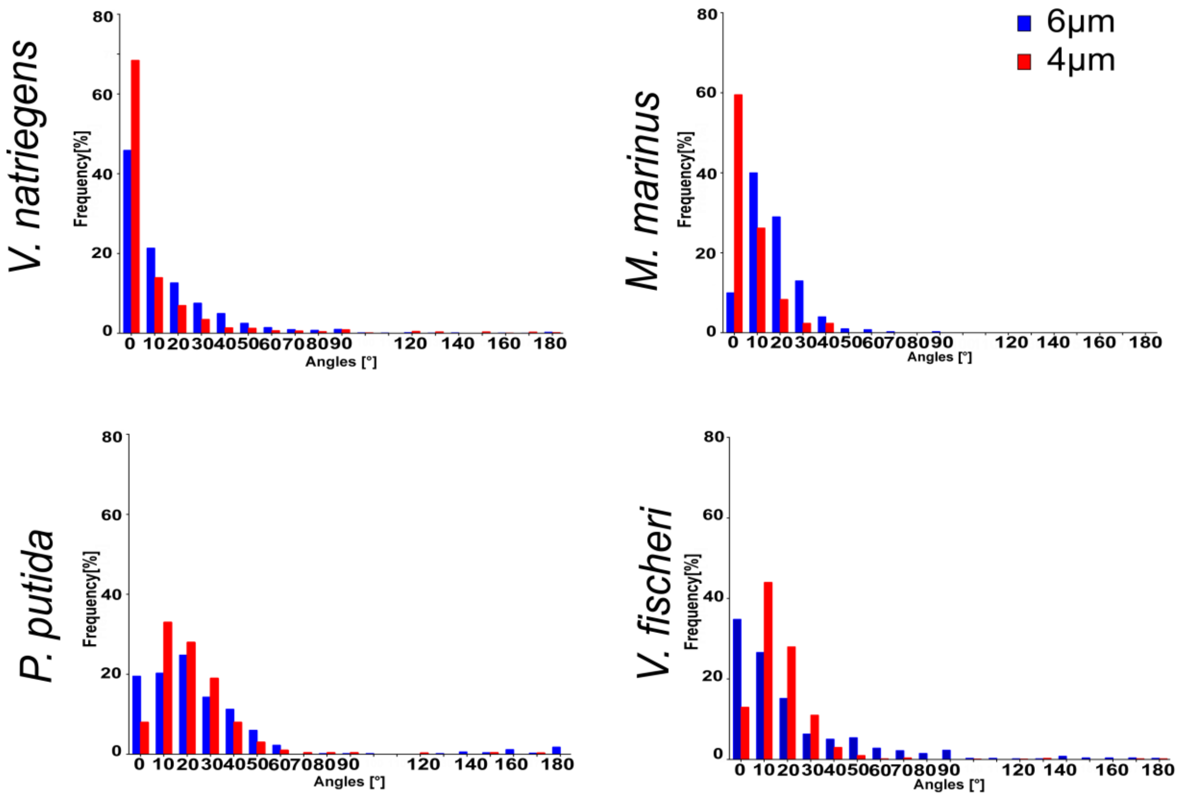

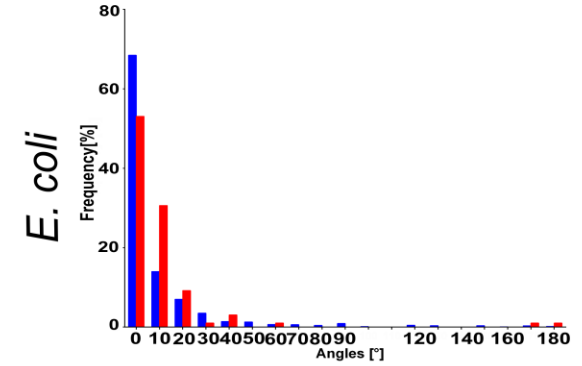

Figure S3. Frequency of the deflection patterns for five different bacterial species, i.e., *V. natriegens*, *M. marinus*, *P. putida*, *V. fischeri*, and *E. coli*, each labelled at the side of the graph, analyzed from the 2D projections of trajectories data collected in the 4 and 6 μm-high plazas.

2.1.1.2. Curvature analysis from 3D trajectories

A finer analysis of the possible coupling of the interaction of the top and bottom horizontal walls of the plazas is possible through the analysis of the representative 3D trajectories. To this end, twenty individual trajectories, each for each plaza with both 6 μm, and 4 μm heights, and for each bacterial species, respectively, were used for *curvature analysis*, with positive values corresponding to clockwise, and negative values corresponding to counterclockwise rotations. The median curvature value was calculated for each trajectory and twenty trajectories of each species and both heights were compared (**Figure S4**), as follows: for parametrized trajectories [x(t),y(t)], the curvature k is

$k(x_0,y_0) = [x_{-1}\cdot y_{-1} – x_1\cdot y_1]/[(x_{-1}^2 + y_{-1}^2)^{3/2}]$, where -1, 0, 1, represent the temporal sequence.

Even a cursory inspection of the distribution of the average curvature for all bacteria showed much streamlined trajectories, that is, the considerably narrower distribution of curvatures, which could be understood only by the constrained applied by both horizontal walls.

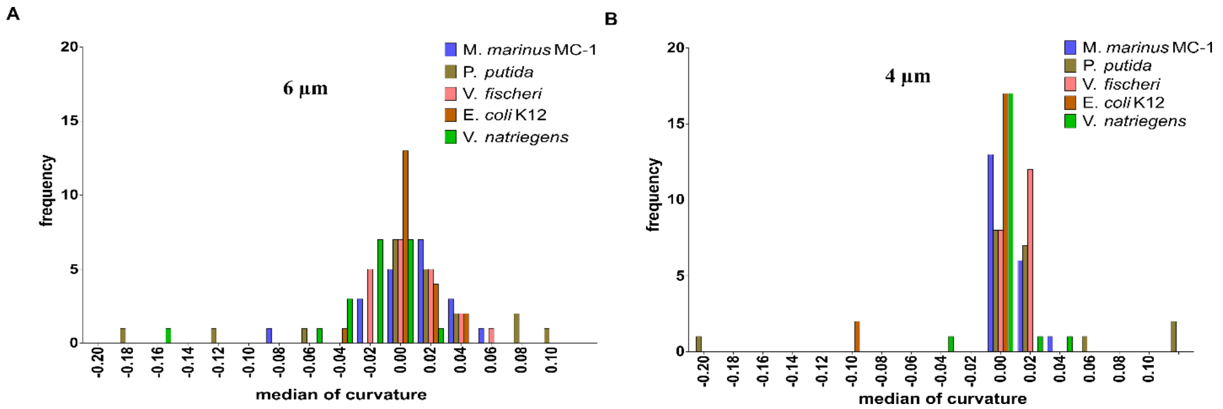

Figure S4. Curvature analysis of trajectories in plazas with 6 µm (A) and 4 µm (B) heights.

2.1.1.3. Preliminary analysis of bacterial motility in 4 µm *tall plazas*

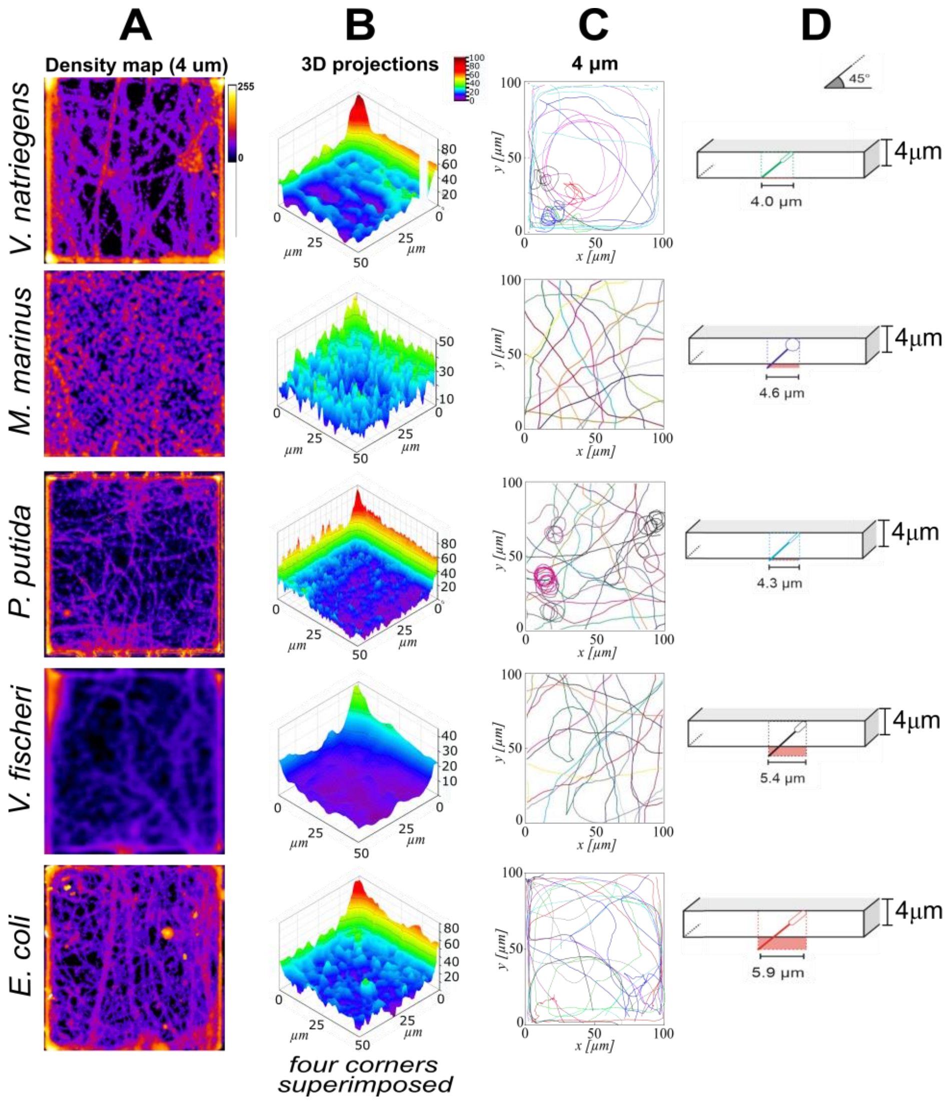

Figure S5. Motility in plazas with 4µm heights. **A**. Density maps of bacterial locations. **B**. Probabilities of bacterial positions. Note the differences in spatial distribution of probabilities compared with those for plazas with 6µm heights**. C.** Characteristic longest trajectories of bacterial motility. Note the differences in trajectory features compared with those for plazas with 6µm heights. **D**. Projection of bacterial length fit across the height of the chamber at 45º.

Detailed explanations for Figure S5: **A**. Density maps of bacterial locations in plazas. Left column; “0 (min)” and “255 (max)” represent the color-coded heat map of no/highest bacterial population, respectively. **B**. Estimation of probabilities of positions, estimated from the 2D projections of the bacterial positions on a horizontal plane, followed by superimposition of the four corners of the density maps of the plaza (similar procedures as in **Figure 2B**, but presented here in 3D). Left column; “0 (blue, min)” and “100 (red, max)” represent the color code for bacterial density along the corners and the walls. **C.** Characteristic longest trajectories of bacterial motility, as 2D projections. **D**. Graphical projection of bacterial length fit across the height of the at 45º. By rows, from top to bottom: *V. natriegens* (average count of bacteria in each frame, n = 14/frame); *M. marinus* (n = 12/frame); *P. putida* (n = 15/frame); *V. fischeri* (n = 15/frame); and *E. coli* (n = 13/frame). Movie S1 presents bacterial overall movement in plazas, and representative trajectories (similar to C). Note the rather considerable difference between the long trajectories (column C) compared with equivalent representation for trajectories in **Figure 2C**.

2.1.2. Overall spatial density.

2.1.2.1. Quantification of bacterial positions at a distance from the horizontal walls in the plazas

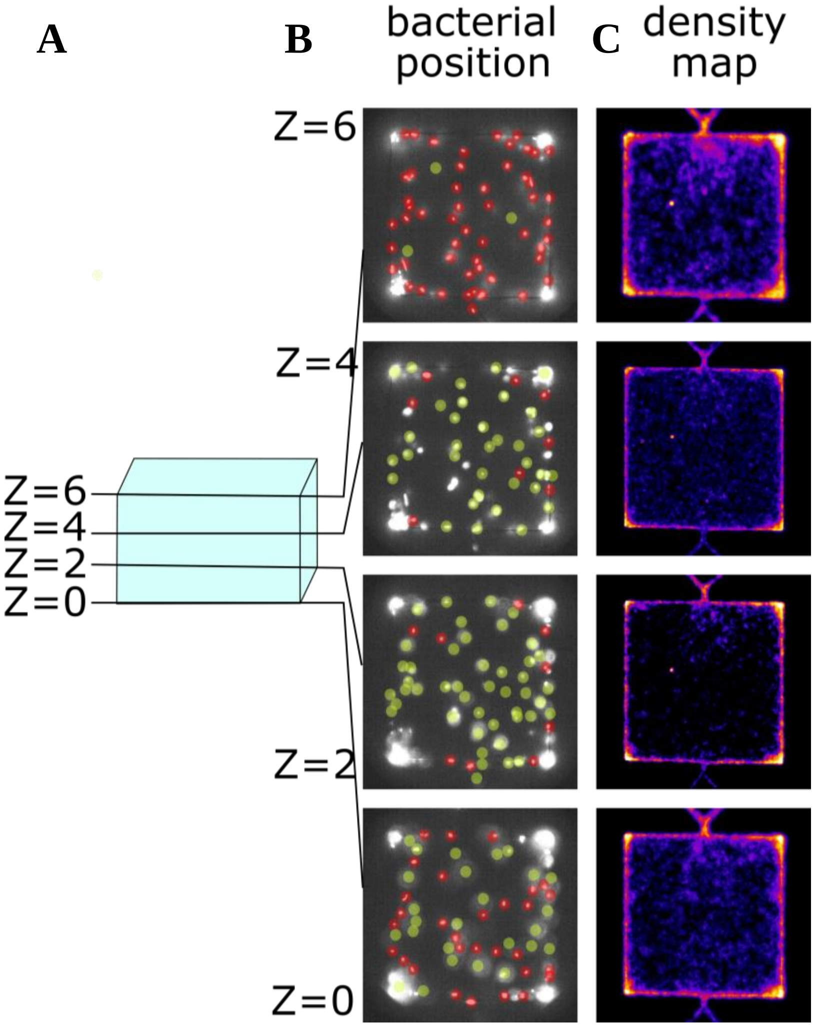

Figure S6. Quantification of bacterial positions using z-stack imaging of the plazas volume (representative analysis for *E. coli*). **A.** Schematic representation of the chamber, with four z-stacked planes, distanced by 2 µm step size. **B**. Assigning of the position of bacteria. The yellow circles over fluorescent signal represent the bacterial species that are out of focal plane, while the red circled overlapped over the fluorescing bacteria represent the bacterial outside the focal planes. **C.** Density maps for the representative z-resolved *E. coli* imaging at different levels. The density maps show that most of bacteria were placed close to the top and bottom horizontal planes.

2.1.2.2. 3D trajectory analysis and z-positioning of five bacterial species in tall plazas.

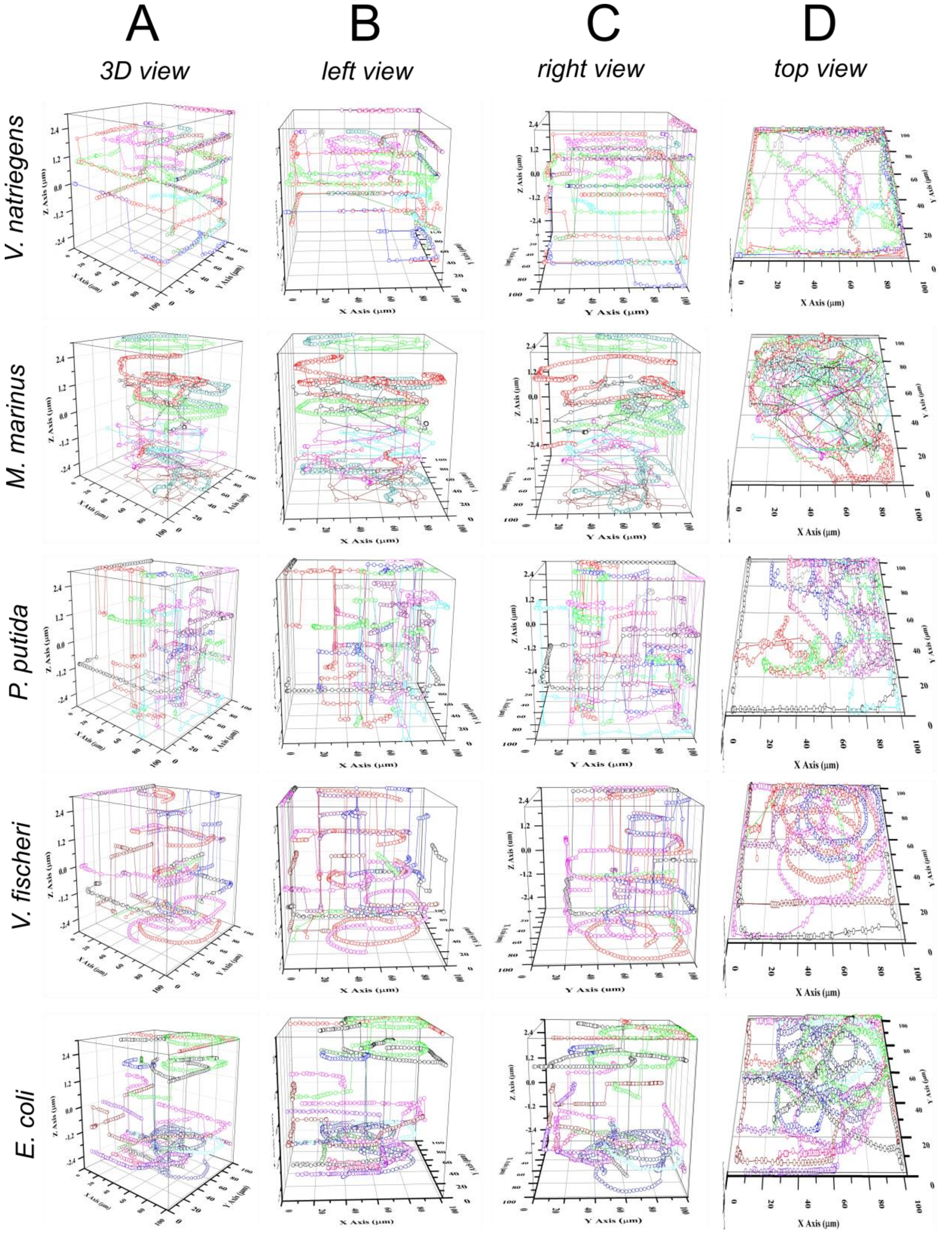

Figure. S7. Representation of bacterial trajectories in 3D using z-stack imaging in plazas. Most of trajectory lengths (density of bacterial positions) were close, and often parallel to the adjacent wall. The transitions between one plane to the opposing one occurred quickly (few points, in few ms). For *M. Marinus* (presenting helical motility patterns) and *P. Putida*, most trajectories were placed away for the horizonal planes, especially in the center for the plaza. Images represent different view angles of the 3D trajectories (complementing **Figure 2D**).

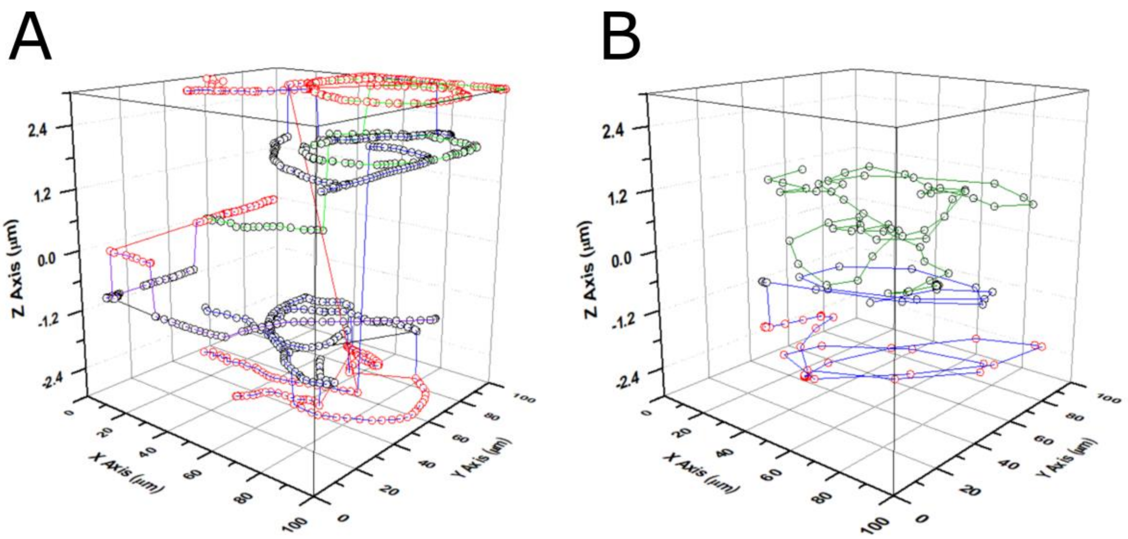

Figure S8. Comparison of two bacterial species with opposite motility behavior. **A**. *E. coli* moves in planes parallel to the walls, at different distances. **B**. *M. marinus* does not move in a parallel plane, thus flipping from a wall accumulator to a wall escaper behavior.

2.1.2.3. Estimation of the distance of swimming parallel to the walls

The first estimation of the distance bacteria swim away from the wall was provided by the "probability map" (**Figure 2B**). This estimation was statistically precise, but it suffered from the edge effects, as the walls the bacteria are swimming near are only 6 μm in width. A fundamentally better (but statistically weaker) option is to collect the z coordinates of the 3D trajectories in the central region of the plazas, i.e., away from edge effects, and then to construct histograms of the bacterial presence away from the horizontal walls (**Figure S9**).

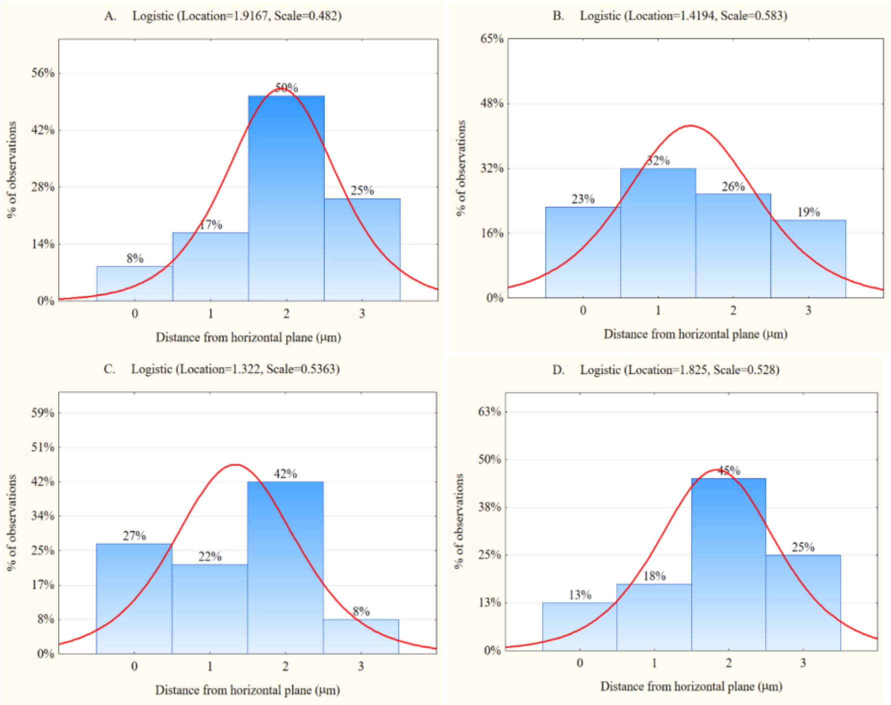

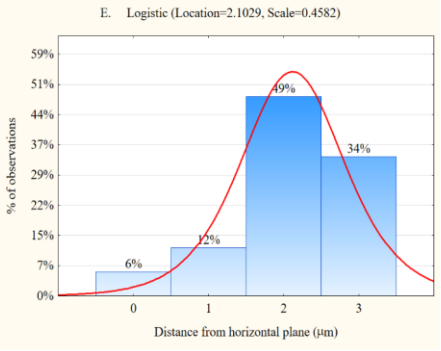

Figure S9. Histograms of the positions of bacteria away from horizontal walls (either ceiling, or floor) of the plazas. From top to bottom: **A.** *V. natriegens;* **B**. *M. marinus;* **C**. *P. putida*; **D.** *V. fischeri*, and **E.** *E. coli*. The logistic fit was used to determine the average bacterial position along vertical axis, and it does not reflect an actual distribution for *M. marinus* and *P. putida*.

2.1.2.4. Simulation of motility of monotrichous bacteria near walls

Numerical results for boundary interactions shown in Figure 3 are upgraded from previous work.(20) The data were generated as follows. A model bacterium is considered consisting of a spheroidal cell body propelled by a single rigidly rotating flagellum. Body and flagellum shapes based on experimental measurements are depicted in Figure 3. The variable $\bar{a}$ is defined as the characteristic length scale of the body. The choice of semi-major axis a_1 and semi-minor axis a_2 of the body result in various aspect ratios a_1/a_2 while maintaining a fixed cell volume $V = (4/3)\pi a_1 a_2^2 = (4/3)\pi \bar{a}^3$. The values of $\bar{a}$ based on cell lengths and widths for the studied bacteria are given in **Table S2**. In addition to the body aspect ratio, the length of the model flagellum is varied, keeping its helical pitch and amplitude fixed.

For each set of geometry parameters, a Boundary Element Method was used to numerically calculate the velocity of the bacterium placed in a given configuration specified by the distance h and orientation angle θ relative to a solid wall. The instantaneous translational and rotational velocities of the bacterium are determined by satisfying the equations of Stokes flow subject to no-slip boundary conditions on the surface of the cell body, flagellum, and wall. The propulsive thrust generated by rotating the flagellum is generally not exactly aligned with the axis of the body, leading to a slight wobbling motion. To determine the average velocity over timescales longer than a motor revolution, the instantaneous velocities was calculated at uniform increments of the angular phase of the flagellum relative to the body and take the mean value. This process allows us to define an average vertical speed $\frac{dh}{dt}$ and rate of turning $\frac{d\theta}{dt}$ that depend on the current h and θ.

For some bacterial shapes, it was found that there is a certain combination of distance and orientation, denoted (h^*, θ^*), at which $\frac{dh}{dt} = 0$ and $\frac{d\theta}{dt} = 0$. This is an equilibrium configuration because the swimmer remains at this height and orientation indefinitely. Moreover, this is a stable equilibrium because bacteria starting at other configurations, provided they are not pointing too sharply away from the surface, would approach the equilibrium point. Bacteria with such an equilibrium point are referred in this study as 'swimmers parallel to wall' because they tend to remain in this plane (which is close to boundaries). This regime is placed at the top right quadrant in Figure 3. The stable configuration was computed for various combinations of the cell body aspect ratio and flagellum length and the stable height h^* was graphically presented by a color scale in the upper right of Figure 3.

As the body aspect ratio decreases (approaching a spherical shape) for a fixed flagellum length, it was found that the stable height decreased until the bacterium became too close to the wall for numerical methods to be reliable. Since these model bacteria also have a strong attraction to walls, in fact descending into walls, they are classified in this study as 'boundary accumulators'. This regime is placed, approximately, at the top left quadrant in Figure 3.

Starting from boundary accumulators with a computed stable configuration, decreasing the flagellum length increases the stable height. There is a region of rapid transition from $h^*/\bar{a} \approx 1$ (where the cell body is very close to the wall) to $h^*/\bar{a} > 5$. Beyond this point, the stable height is far enough from the wall that hydrodynamic interactions are negligible. In this regime, located in the lower portion of Figure 3, the bacteria turn and swim away from the wall even if they are initially approaching the wall, thus referred 'boundary escapers'.

The hydrodynamic explanation for why changes in the geometry of the bacterium affect its motion near walls is that the shape determines the distribution of stresses acting on the cell membrane and flagellum. The flow field generated by a swimming bacterium can be

approximated by combinations of a force dipole, source dipole, and higher order terms that each produce different interactions with a wall.(21) The relative strengths of these terms, and hence the net behavior near walls, depend on the shape of the organism.

Table S2. Characteristic geometrical parameters of the bacteria studied used for the simulation of motility behavior (monotrichous architecture model), as in Figure 3.

**Parameter→ Bacteria↓**	**a_1/a_2**	**L/ā**	**h*/ā**	**$L+a_1$ (µm)**
V. natriegens	1.75 ±0.75	9.51 ±3.48	3.05	5.45 ± 2.30
M. marinus	10.2 ±0.21	4.35 ±1.30	1.35	4.95 ± 1.80
P. putida	3.19 ±1.56	10.84 ±5.03	2.47	4.70 ± 1.90
V. fischeri	3.11 ±0.69	12.34 ±4.15	3.73	6.00 ± 3.40
E. coli	2.10 ±1.02	8.64 ±4.33	2.74	5.65 ± 2.30

Legend (32):
a_1 = polar radius of cell body (half the cell length); a_2 = equatorial radius of cell body (half of the diameter diameter); [a_1/a_2] = aspect ratio of the cell body; L = curvilinear length of the flagellum (approximated by the axial length of the flagellum); ā = radius of sphere with volume of cell body; L/ ā = aspect ratio of the bacterium; h* = optimal distance from the wall (for swimmers parallel to the walls), determined in this study from the z-stack analysis (**Figure S9**); and h*/ā = non-dimensional distance from the wall.

2.2. Motility in tightly confining geometries

2.2.1. Motility in linear channels

2.2.1.1. Overall motility characteristics

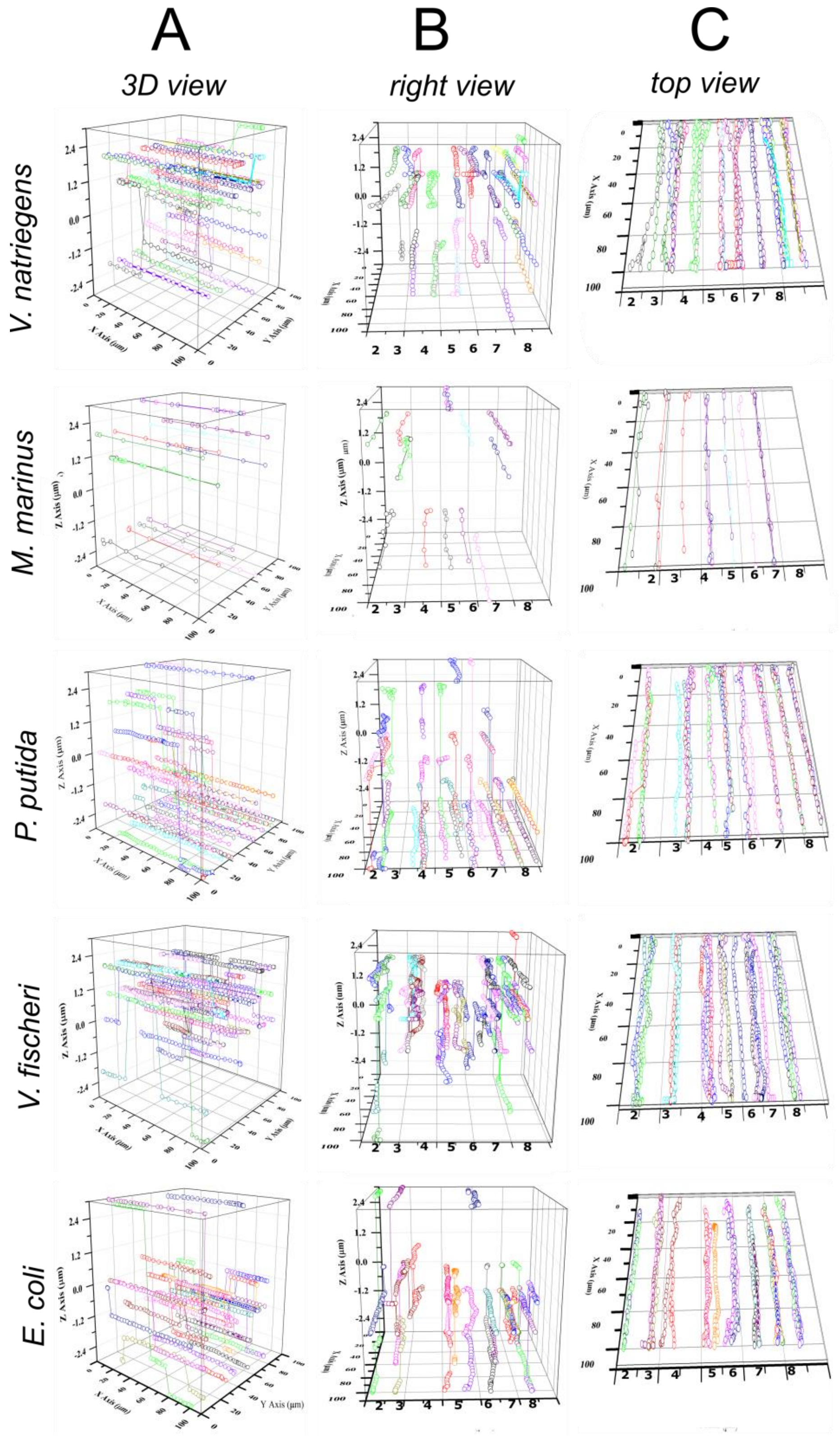

Figure. S10. Cumulative representation of trajectories of five different bacterial species projected in 3D space using z-stack imaging in linear channels.

2.2.1.2. *Analysis of the possible sinusoidal motion of bacteria in straight channels*

2.2.1.2.1. *Detecting potential helical motion from 2-dimensional camera data with multiple waveforms*

We consider the general problem of determining, from 2D tracking data, whether a bacterium is executing 3D helical motion or 2D, planar "sinusoidal" motion. To do this, we aim to convert 2D data to three dimensions, under the assumption the helical motion is taking place. This then allows us to essentially test the hypothesis of helical motion.

We begin by considering the directional components of the bacterial velocity, derived from positional data. Given a 2D track, we can compute $v_x(t)$ and $v_y(t)$, i.e., the x and y components, as a function of time, while $v_z(t)$, the z-component, is hidden from us due to the use of a point of view placed orthogonally to this axis. Given $v_x(t)$ and $v_y(t)$, our specific goal is to estimate the unobserved velocity component v_z.

Observe that the (unknown) magnitude of the total velocity is given by

$$V(t) = \sqrt{v_x(t)^2 + v_y(t)^2 + v_z(t)^2}$$

At any point where the bacterium is moving orthogonal to the axis of the camera, we have $v_z = 0$, such that the total velocity follows

$$V_\perp = \sqrt{v_x(t)^2 + v_y(t)^2}$$

We now assume (but see below) that the movement of the bacterium has no net drift in the z-direction. This implies the bacterium is moving orthogonal to the camera axis twice in each spiral of its 3D helix.

The total magnitude of velocity can be estimated as a smooth curve fit over the peaks of the $V_\perp$ curve, for instance using spline interpolation. At each peak, $v_z = 0$ and its sign alternates as the helically moving bacterium completes each corkscrew. This allows the estimation of v_z at all other points.

This approach has limitations, because of the loss of information from 3D information being projected into 2D. If the bacterium is moving toward or away from the camera for significant portions of its trajectory, our method is unable to estimate its total v_z component, absent further assumptions about the specific pattern of swimming. In addition, this estimation procedure is inaccurate if the trajectory is not close to a symmetrical, repeatable helix, e.g., if the helical motion is highly stochastic. The technique is inaccurate in the absence of a sufficiently high number of data points, allowing a smooth spline to fit to be applied to the $V_\perp = \sqrt{v_x(t)^2 + v_y(t)^2}$ curve. Lastly, it is not possible, by this approach, to determine the "handedness" of the helix. However, we note that for many bacterial species, the handedness is known or can be determined experimentally, because it is genetically controlled.

Perhaps most crucially, however, this method requires the available tracking data from the camera to contain multiple peaks and troughs of the trajectory, otherwise the spline fitting to the peaks of the $V_\perp$ curve is not possible. For situations where only one or less peak-to-trough sections are available, a different approach is needed.

2.2.1.2.2. Detecting potential helical bacterial motion from 2D camera data where only fragments of sinusoidal tracks are present

In this situation, it is possible to estimate the wavelength of the sinusoidal motion from fragments containing one or two peak-to-trough sections by employing a Fast Fourier Transform (FFT) analysis. Specifically, the FFT spectrum of the *x-y* curve (in 2D) will exhibit one dominant frequency if sinusoidal (potentially resulting from helical motion projected two-dimensionally) is present; otherwise, the spectrum will be highly noisy and/or flat.

We ran all tracks in different channel widths for all five bacterial species through an FFT analysis, leading to the ability to directly fit sine curves to the *x-y* trajectories and estimate the wavelength of the motion in the channel. This approach is illustrated (using a randomly chosen bacterial track from the data set) in Figure X.1 below, in which the right hand side panel shows the results of the fitting procedure and the left hand panel shows the FFT spectrum, exhibiting a dominant frequency. From this, the motion wavelength can be estimated as the inverse of the dominant frequency (code available on request).

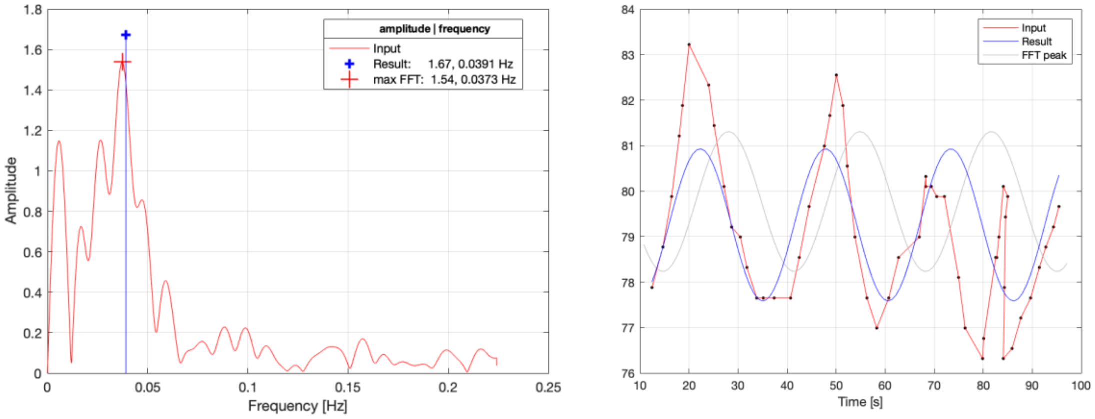

Figure S11. Left: FFT analysis of a bacterial track in a channel, allowing the identification of the frequency of the sinusoidal motion (and thereby the wavelength) as the peak of the FFT spectrum (blue line and red cross). Right: in red, the bacterial track projected as an x-y trajectory; in blue and gray, the resulting sine curve fits corresponding to the FFT peak frequency (gray) and average weighted frequency (blue).

2.2.1.2.3. Estimation of sinusoidal characteristics of bacterial motility in straight channels

We used this method to study the variation in sinusoidal movement (resulting from 3D helical movement) of different bacterial species in different channel widths from 2μm to 8μm. The results are shown in **Figure S12C**.

The sinusoidal character of the motion of *P. putida* and *V. natriegens* varies strongly with the widths of the linear channels, possibly due their shortest body lengths of all species studied. The increase of the wavelength of motion with the increase of the channel widths is probably due to the larger volume available for 3D helical movement. It is notable that a sinusoidal movement has been predicted(22) for monotrichous bacteria (such as *V. natriegens*) and for wall escapers (as partially exhibited by *P. putida*, **Figure 3**).

At the other end of the spectrum, *M. marinus*, with its frequent collisions with and bouncing from the walls, appeared to be insensitive to channel widths with regard to a sinusoidal character of movement.

Finally, in a medium class, *V. fischeri* and especially *E. coli* appear to have only a modest evolution of the sinusoidal character of movement with the increase of the channel widths, possibly due to their longest body lengths in all species studied.

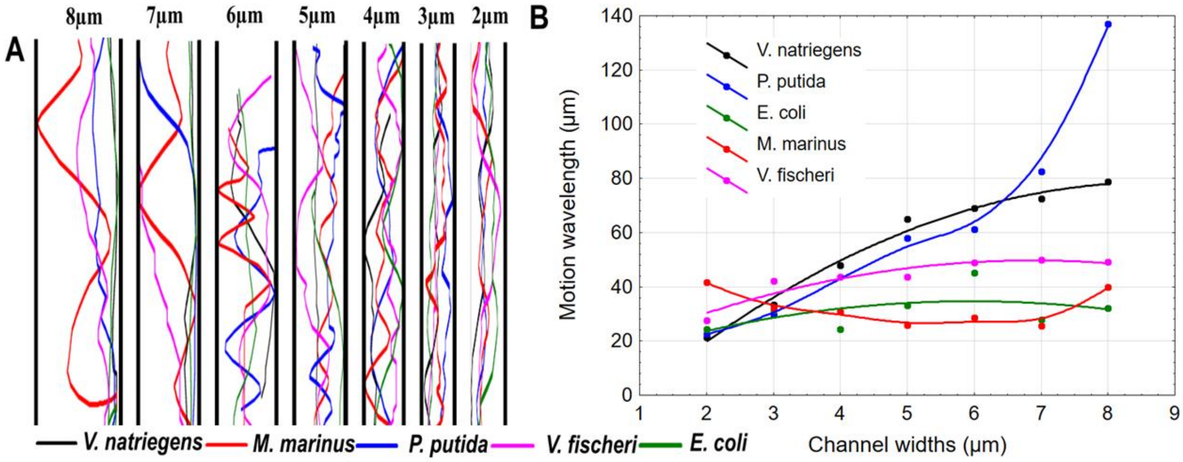

Figure S12. Representative trajectories in the linear channels (A, left); and the variation of the estimated motion wavelengths versus channel widths (B, right).

2.2.1.3. *Velocity in straight channels*

To assess the impact of confinement on bacterial velocity, the average velocity of bacteria was measured, in straight channels, and in plazas, the latter seen as straight channels with 100 µm widths (**Figure S13**).

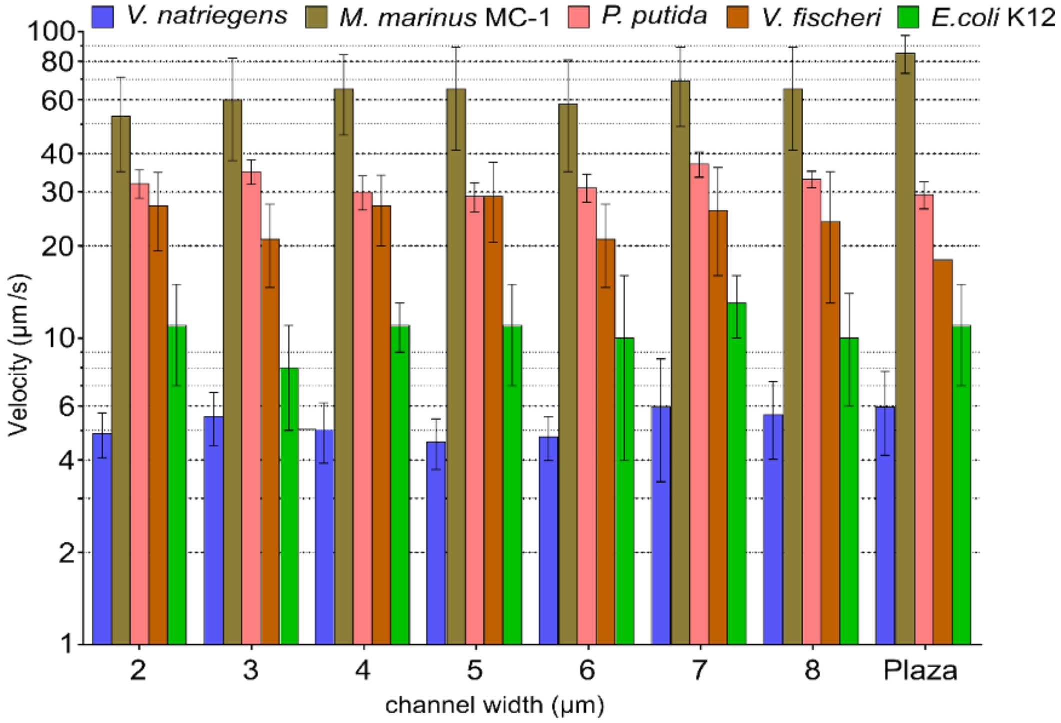

Figure S13. Bacterial velocities (logarithmic scale) in straight channels and plazas.

A moderate decrease was observed for the velocity of *M. marinus*, assumed to be a result of the amplification of the collisions with the walls, but for all other species the velocity does not vary importantly with the channel width.

This observation is further substantiated by the more precise and more detailed measurements of velocities offered by double histograms of velocities in channels (**Figure S14**). Also, with the exception of *M. marinus*, for which extreme narrow channel 'force' motility at the walls, and *V. natriegens* and *E coli,* presenting a bimodal distribution of velocities, towards the channel center, and again a secondary bimodal distribution at the walls (as presented above), all other species behave as swimming parallel to the walls, that is, near the center of the channels, as predicted by **Figure 3**.

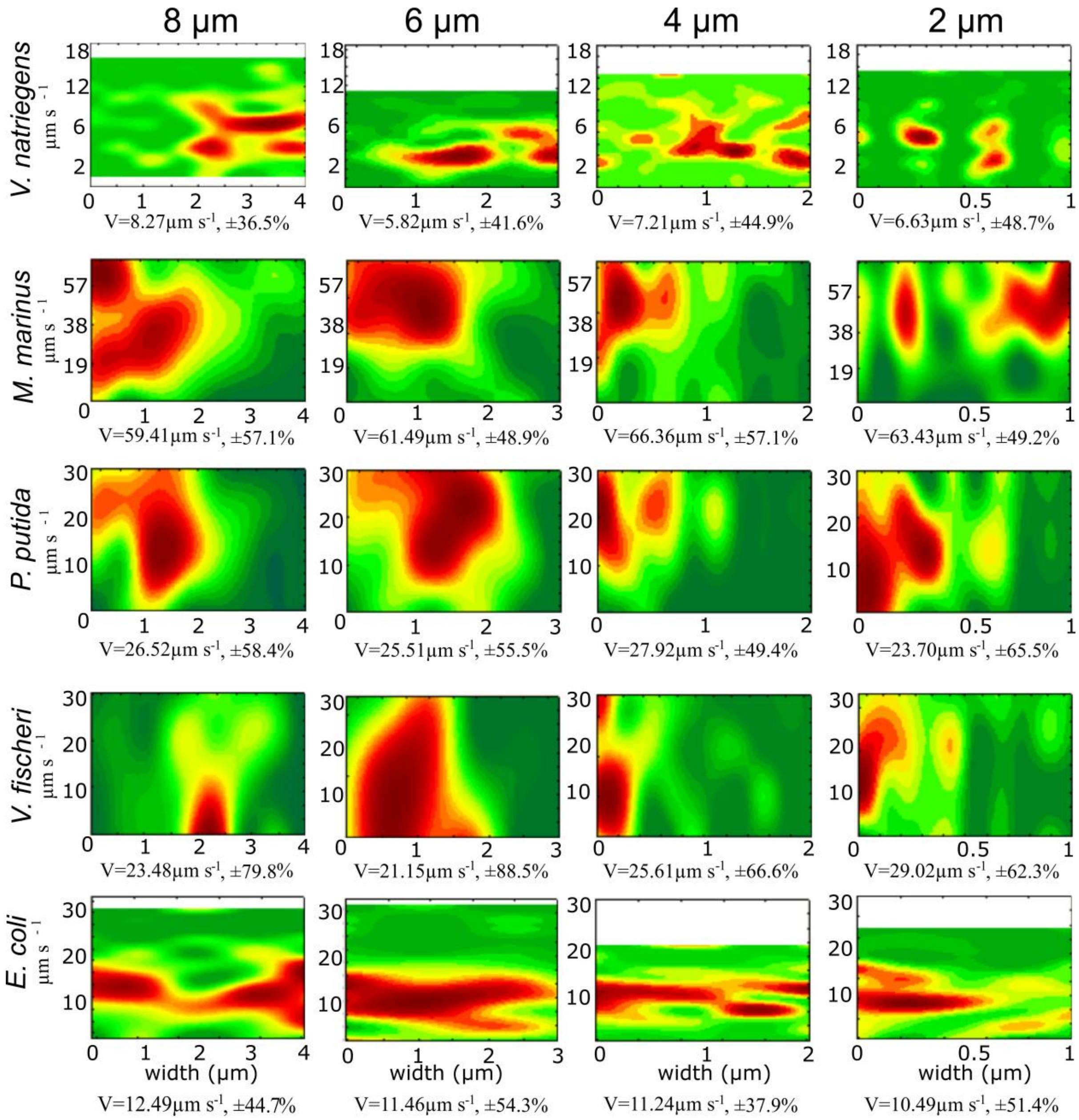

Figure S14. Double histograms of bacterial velocities in linear channels with 2 μm, 4 μm, 6 μm, and 8 μm widths. All velocities values are normalized, i.e., top y-axis value is the maximum. The channel widths are also normalized, i.e., left value on the x-axis represents the channel center, and the right value represents the wall. Note the resilient bimodal distribution, at the walls, for both *V. natriegens* and *E. coli*.

2.3. Motility in channels with angled exits

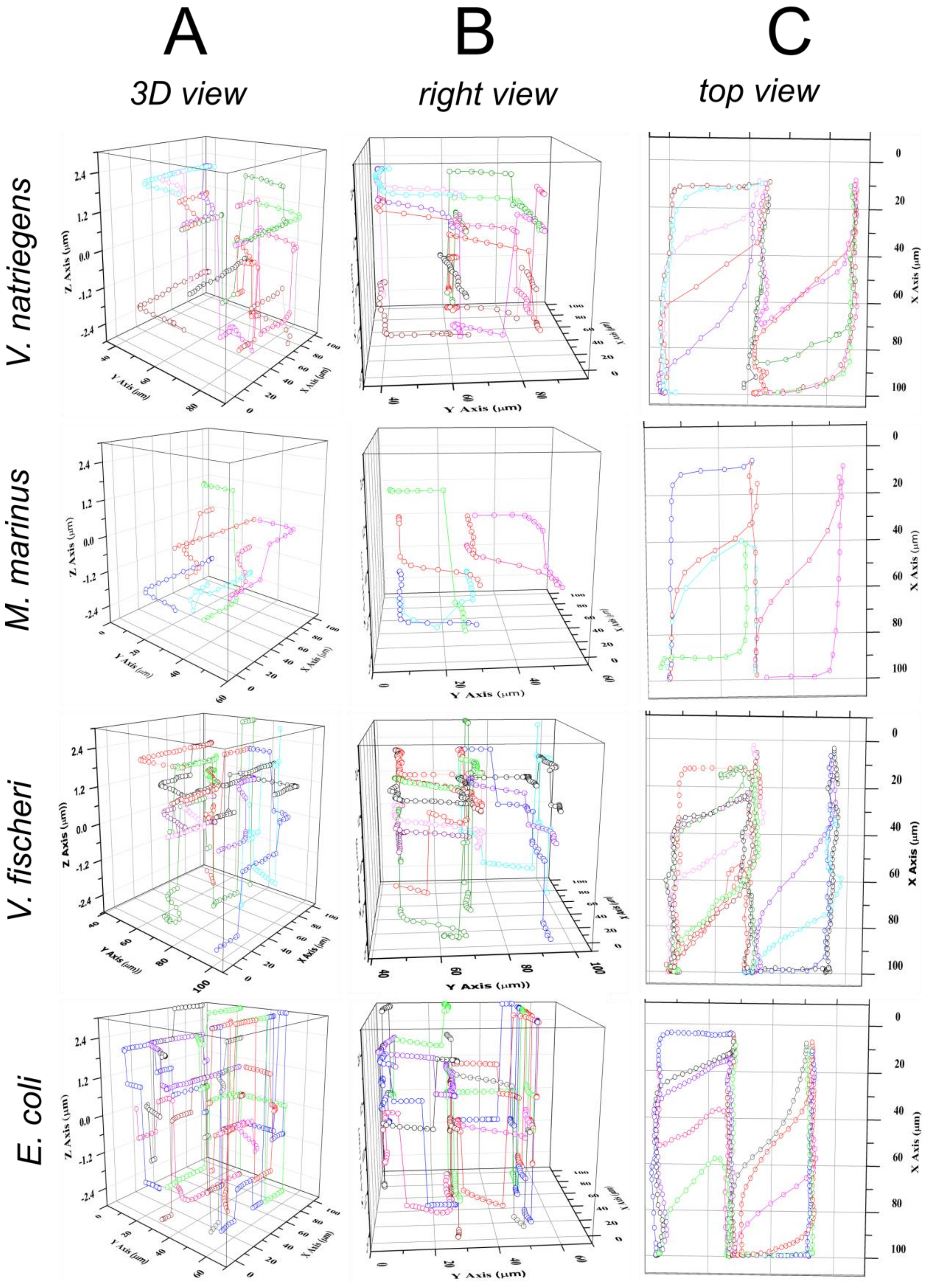

Figure. S15. 3D trajectories of five different bacterial species projected in 3D space using z-stack imaging in angled channels.

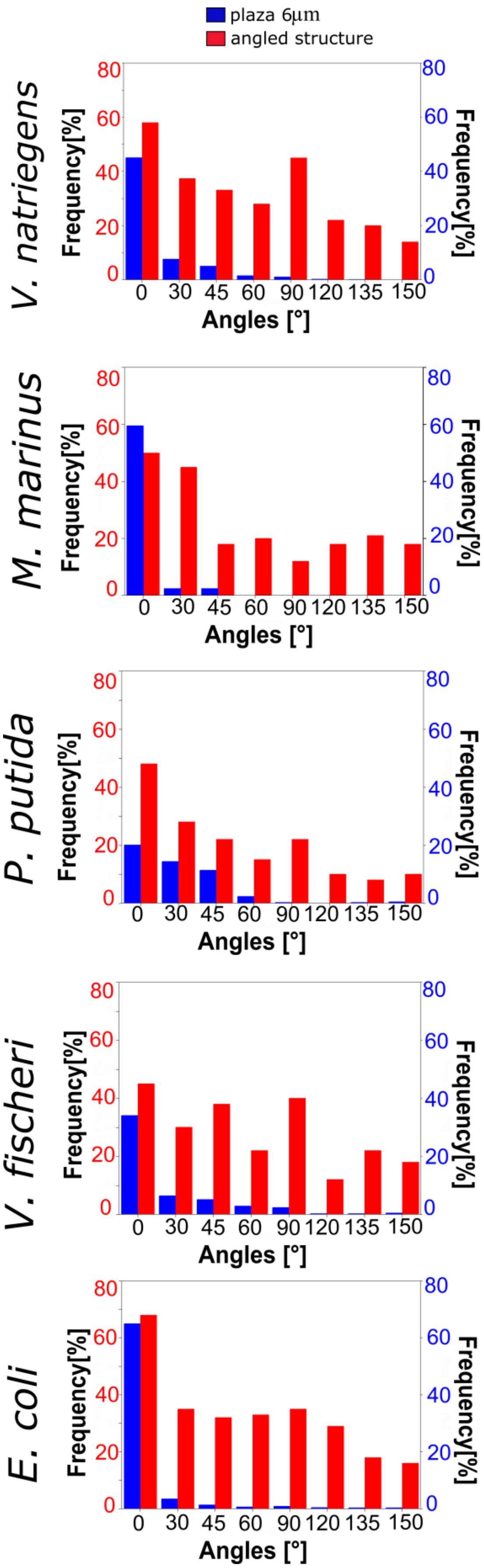

Figure. S16. 3D trajectories of five different bacterial species projected in 3D space using z-stack imaging in angled channels.

2.3.1. Motility in meandered channels

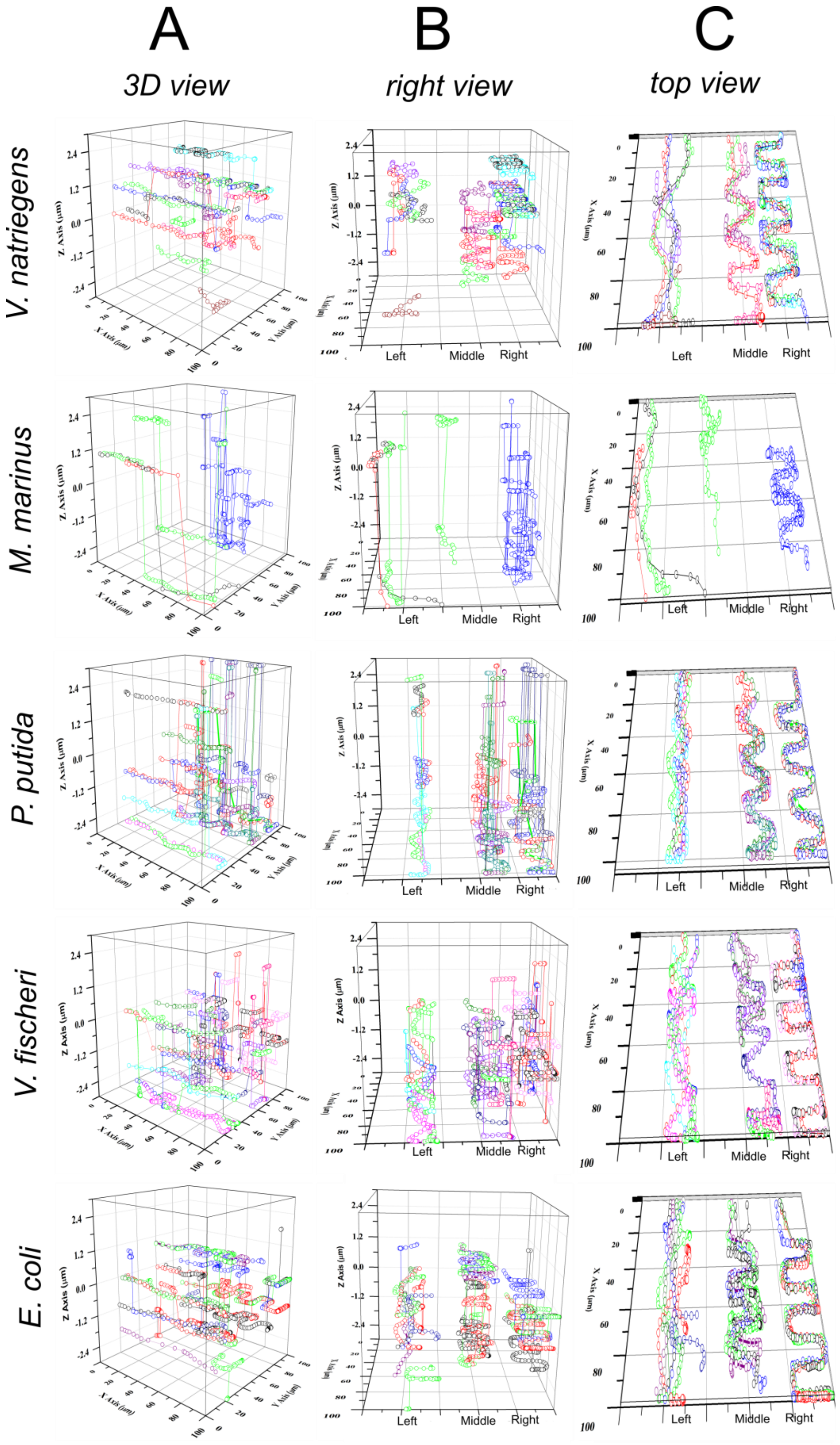

Figure. S17. 3D trajectories of five different bacterial species in meandered channels.

Table S3. Comparison of the average time-spent (seconds) by bacteria in meandered channels with different tooth widths (left-middle-right channels as in **Figure S2C**).

**Meandered channels:**<br>**Bacteria↓ Tooth length→**	**Left**<br>5 µm	**Middle**<br>10 µm	**Right**<br>15 µm
V. natriegens	4.5 ±1.8	24.0 ±10.2	27.5 ±7.3
M. marinus	3.2 ±2.0	5.5 ±3.3	7.3 ±3.2
P. putida	7.9 ±5.1	11.2 ±4.4	15.4 ±5.6
V. fischeri	9.9 ±5.8	49.1 ±8.9	38.8 ±16.3
E. coli	24.3 ±19.3	43.1 ±14.9	65.9 ±22.3

Table S4: Comparison of the average time-spent (seconds) by bacteria that succeeded in traversing the meandered channels with different tooth widths (left-middle-right channels as in **Figure S2C**).

**Meandered channels**<br>**Bacteria↓ Tooth length→**	**Average time spent in succeeding the trapping (seconds)**		
	**Left**<br>5 µm	**Middle**<br>10 µm	**Right**<br>15 µm
V. natriegens	5 ±1.8	NA	28 ±7.4
M. marinus	3 ±2.0	5 ±3.3	7.5 ±3
P. putida	7.5 ±5	11.8±4.4	15.4 ±5.6
V. fischeri	10 ±5.6	38.1 ±8	38.8 ±16.3
E. coli	20.5±18	NA	30 ±22.3

Note that bacterial species like *V. natriegens* and *E. coli* has a zero or negligible success rate in traversing the middle-sized meandered channel during experimental observation.

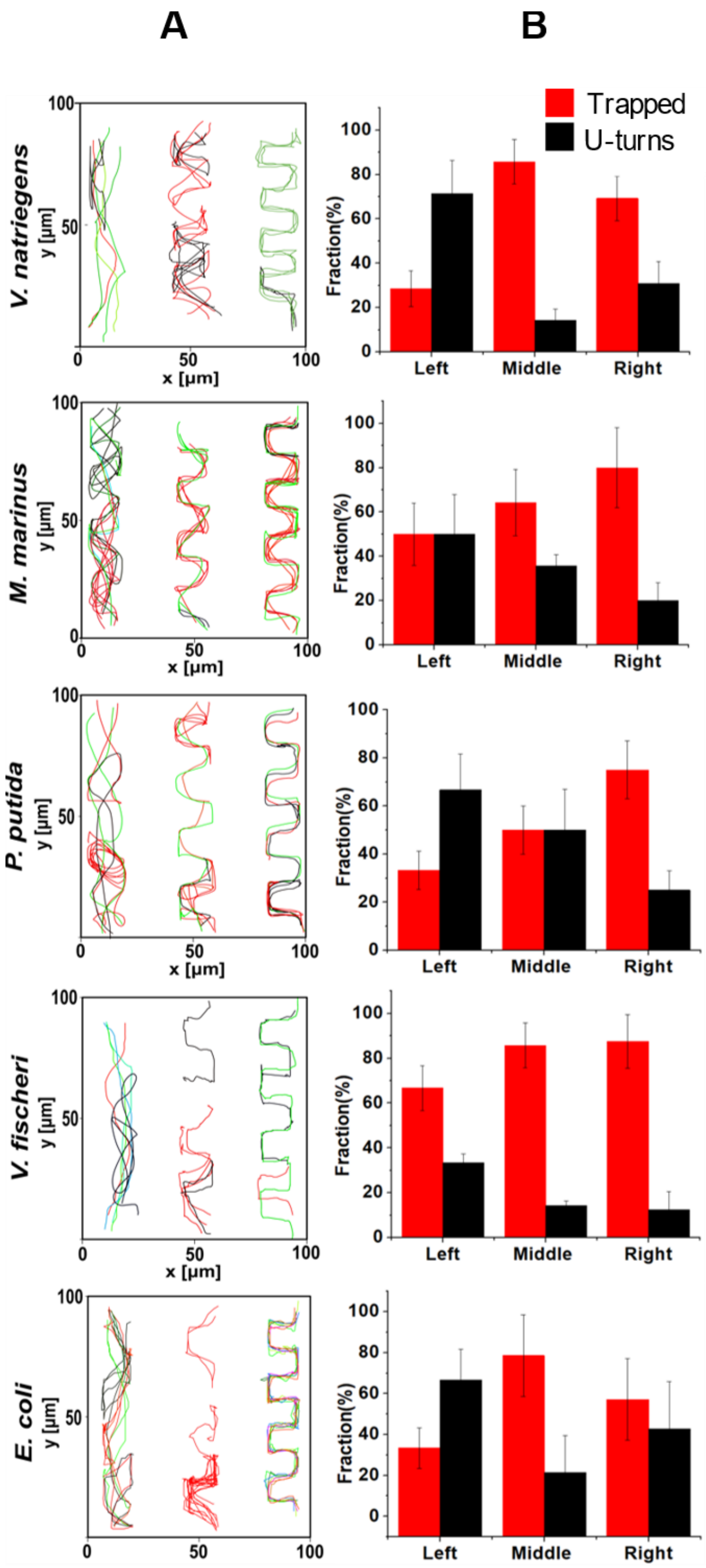

8

Figure S18. Bacterial motility in meandered, comb-like channels. **A**. Representative tracks of the bacterial motility: red trajectories - bacteria took U-turns; black trajectories - bacteria got trapped; any other color - bacteria successfully traversed. **B**. Distribution of unsuccessful bacteria that made either a U-turn or are trapped. By rows, from top to bottom: *V. natriegens* (average count of bacteria each frame, n = 18/frame); *M. marinus* (n = 12/frame); *P. putida* (n = 22/frame); *V. fischeri* (n = 25/frame); and *E. coli* (n = 19/frame).

2.3.4. Velocity distribution for bacterial species in different motility structures –

The velocity observed in various microfluidics structures were expected to be related to the respective levels of confinement, with the highest confinement levels present in the meandered channels (“combs” in **Figure S17**); and the lowest in plazas. However, while the variation of velocity due to confinement were obvious for *M. marinus*, it is far less obvious for other species. However, among the different structures studied here, the motility in the meandered channels present the most noticeable decrease of apparent velocity (with the notable exception of *V. natriegens*). For instance, *E. coli* and *V. fischeri* decreased their velocities by more than 75% compared to its velocity in plazas, and P. putida with almost 50%, This behavior is easily understood if connected with the frequent trapping in the corners of the meandered channels, reducing its overall average velocity. Such steep changes were not observed in the other species, suggesting there was not a strong corner preference for rest of the members of bacteria tested. The velocity differences observed with *E. coli* and *M. marinus* are classical examples of steric interactions-based confinement where *M. marinus* was restricted with space leading to reduction in velocity while for *E. coli* it was the geometrical preferences. Finally, it should be noticed that *V. natriegens* is the species with the smallest aspect ratio of the dimension of cell body, and one the species with the lowest ratio of length/cell diameter (**Figure 3**) which suggest an easier negotiation of convoluted geometries.

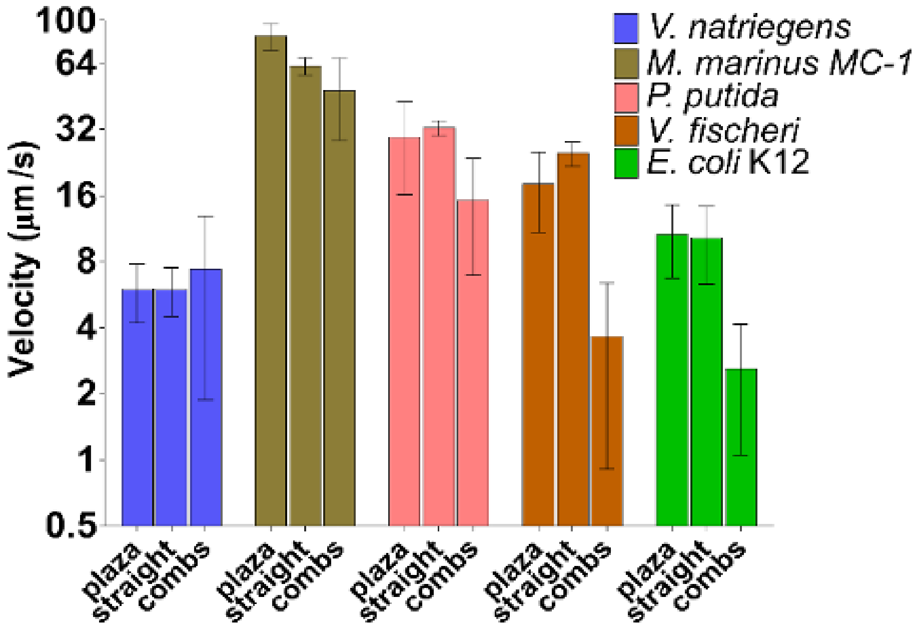

Figure S19. Comparison of bacterial velocities (logarithmic scale) in plazas, straight channels (“straight” label) and meandered channels (“combs” label).

2.8. Tight geometrical like confinements from nature

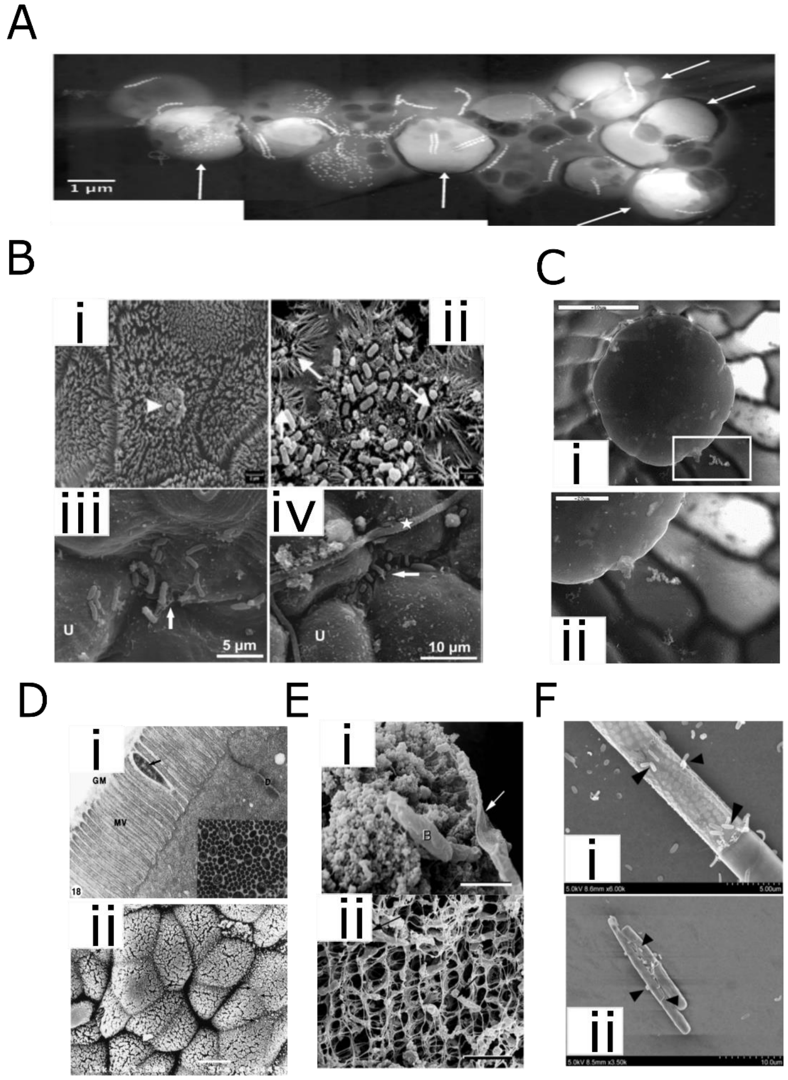

Figure S20. Closer look into the microbial habitats and tight confinements in nature. The above images are adopted from several reported communications, which shows high definition microenvironment that are bacterial habitats and the tight confinements that are common observations among these figures. **A.** *Magnetotactic species* in the soil sediments of lake (23, 24), spherical bacteria in the constrained environments. **B**. *Escherichia species* in the gut (B I & ii) and on plant surfaces (25) (B iii & iv) . The image shows constricted patterns with tight channel like features and different turn angles (iii & iv). **C**. Rod shaped bacterial species (26) . The image shows several straight and different turn angles, like the feature that we used in the devices for studying angled preferences. **D**. *Vibrio species* in the gut of fishes (27). The image D(i) shows straight channel like feature present in the microvilli (MV) and image D(ii) shows different angled and zig-zag patterns, like the structures with higher complexity that we explored in our studies. **E**. *Pseudomonas species* in soil sediments (i &ii) shows features with different pore sizes and highly complex structures (28). **F**. rod shaped bacterium on surface of phytoplankton (29). The figure shows straight lines with many turn angles and most notably 90° turn angles.

Supporting Movies

The movies present typical motility patterns of all bacterial species studied. *E. coli* and *V. natriegens* were labelled with fluorescent expressing plasmids (either GFP or mCherry) and the movies were recorded with a fluorescence microscope. Other species were observed in bright field (all species) or in dark field (*M. marinus* only). All the movies are played at original speed to show different behaviors like the velocity differences among the different species, U-turn types, wall-accumulating and wall repelling motions, circular, chaotic trajectories etc.

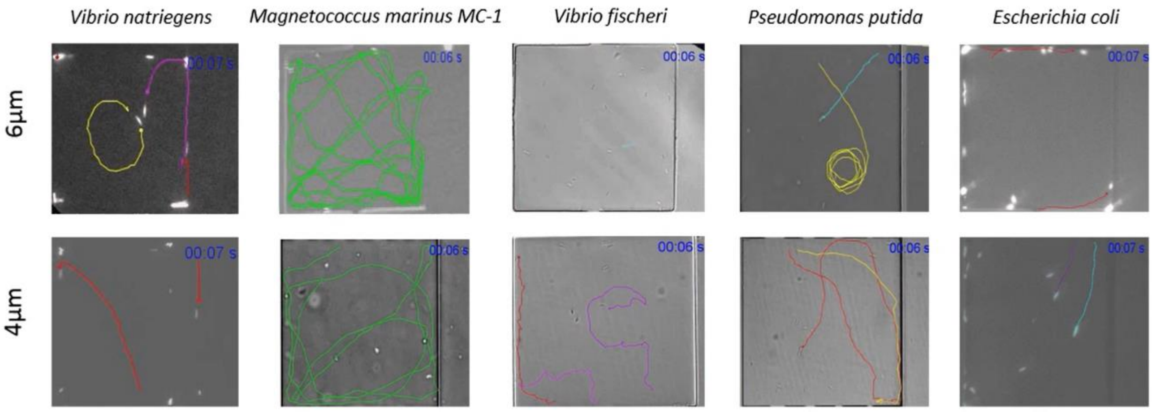

Supporting Movie 1 – Bacterial motility in quasi open spaces (plazas) of two different heights

Trajectories along the walls: Red, Ping-Pong like trajectories: Green, Circular trajectories (longest): Purple, Circular trajectories (Smallest): Yellow, Random trajectories: Cyan

Movie S1. Motility in quasi-open spaces (plazas). Bacterial specific motility observed from the movies and discussed in the main text – Screen shots of the videos is presented here, while the actual movies can be downloaded.

V. natriegens: Preference towards the corners and wall-directed motility. *M. marinus:* Wall-bouncing, ping-pong ball like motility pattern. Restricted motility and few straight trajectories in 4 µm tall plazas, as an effect of confinement. *V. fischeri:* Wiggling, chaotic motion with frequent pauses and change of directions. Observable frequently in 4 µm low plazas. *P. putida:* Typical circular motions with high deflection angles. The diameter of the circular motion decreases due to vertical confinement in 4 µm low plazas. *E. coli:* Wall-dependent motility with corner preferences. Also, small circular motions observed in both high and low plazas.

Linear channels

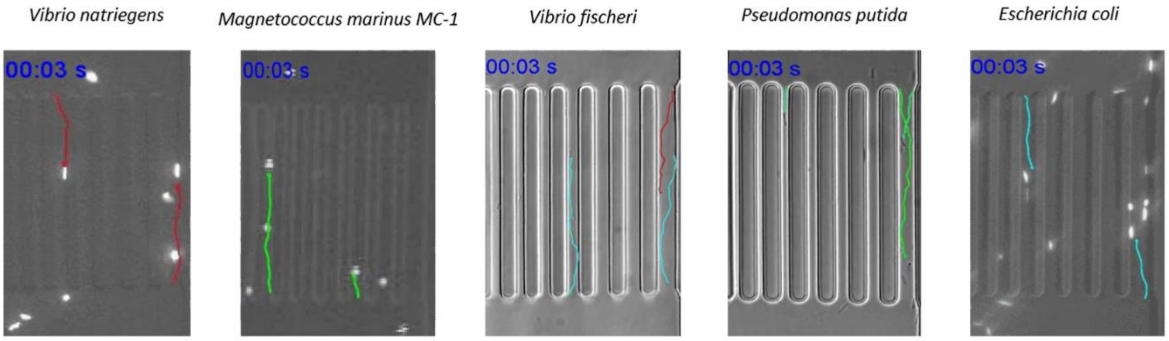

Supporting Movie 2 – Bacterial motility in linear channels

Trajectories along the walls: Red, Sinusoidal like trajectories: Green, Random trajectories: Cyan

Movie S2. Bacterial motility in linear channels. *V. natriegens:* Lowest U-turn frequency with high accumulating. *M. marinus:* High U-turn frequency in narrow channels due to repeated sinusoidal (bouncing) motility and deflection from the walls. *V. fischeri:* Chaotic motility pattern with considerable U-turn frequency, irrespective of the channel dimensions. *P. putida:* Sinusoidal (bouncing) motility and deflection from the walls. *E. coli:* Second highest propensity for wall-accumulating and a low U-turn frequency.

Angled channels

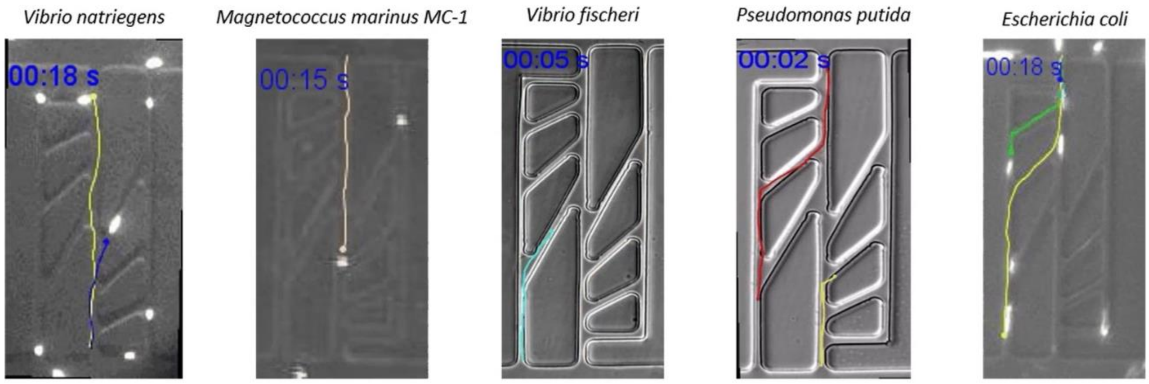

Supporting Movie 3 – Bacterial motility in angled channels

Movie S3. Bacterial motility in angled channels. *V. natriegens:* Preference for moving on straight trajectories along the middle axis channel. *M. marinus:* Trajectories deflected at angles > 135°. *V. fischeri:* Higher U-turn % and partly wall-accumulating behavior at angled channels. *P. putida:* Trajectories deflected at angles > 135°. *E. coli*: Preference for moving on straight trajectories along the middle axis channel.

Combed channels

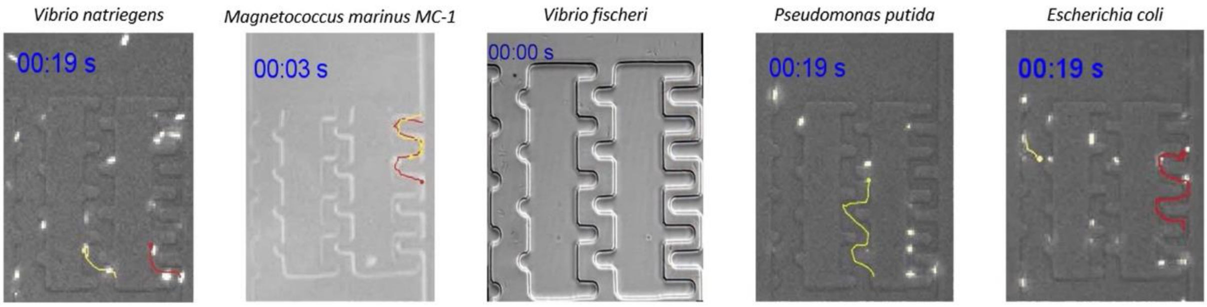

Supporting Movie 4 – Bacterial motility in combed structures

Successful trajectories: Red, Unsuccessful trajectories: Yellow

Movie S4 - Bacterial motility in combed structures. *V. natriegens:* Successfully traversed the combs of lowest and highest tooth length, but not the middle combed-structures due to trapping at the corners. *M. marinus*: Successfully traversed the middle tooth structures compared to the other two combed structures. Least success rate in combs. *V. fischeri:* Possessed a corner–to-corner motility pattern, providing a higher success rates in traversing in all the three comb types. *P. putida:* Exhibited a shifted propensity to follow walls for traversing the combs. Still a second lowest success rate in navigating the complicated comb structures. *E. coli:* 90°: angled corners appear to operate as traps, for *E. coli* very efficiently in middle sized tooth, while the two other combs were successfully traversed.

References for Supporting Information Section

1. S. Cheng *et al.*, Microscopical observation of the marine bacterium Vibrio natriegeus growth on metallic corrosion. *J Materials, Manufacturing Processes* **25**, 293-297 (2010).
2. M. T. Weinstock, E. D. Hesek, C. M. Wilson, D. G. Gibson, Vibrio natriegens as a fast-growing host for molecular biology. *Nature Methods* **13**, 849 (2016).
3. D. A. Bazylinski *et al.*, Magnetococcus marinus gen. nov., sp. nov., a marine, magnetotactic bacterium that represents a novel lineage (Magnetococcaceae fam. nov., Magnetococcales ord. nov.) at the base of the Alphaproteobacteria. *Int J Syst Evol Microbiol* **63**, 801-808 (2013).
4. S. Taherkhani, M. Mohammadi, J. Daoud, S. Martel, M. Tabrizian, Covalent binding of nanoliposomes to the surface of magnetotactic bacteria for the synthesis of self-propelled therapeutic agents. *ACS Nano* **8**, 5049-5060 (2014).
5. M. L. Davis, L. C. Mounteer, L. K. Stevens, C. D. Miller, A. Zhou, 2D motility tracking of Pseudomonas putida KT2440 in growth phases using video microscopy. *J Biosci Bioeng* **111**, 605-611 (2011).
6. J. W. Barton, R. M. Ford, Determination of effective transport coefficients for bacterial migration in sand columns. *Appl Environ Microbiol* **61**, 3329-3335 (1995).
7. M. S. Hendrie, W. Hodgkiss, J. M. Shewan, Proposal that Vibrio marinus (Russell 1891) Ford 1927 be Amalgamated with Vibrio fischeri (Beijerinck 1889) Lehmann and Neumann 1896. *International Journal of Systematic and Evolutionary Microbiology* **21**, 217-221 (1971).
8. D. S. Millikan, E. G. Ruby, Alterations in Vibrio fischeri motility correlate with a delay in symbiosis initiation and are associated with additional symbiotic colonization defects. *Appl Environ Microbiol* **68**, 2519-2528 (2002).
9. M. Binz, A. P. Lee, C. Edwards, D. V. Nicolau, Motility of bacteria in microfluidic structures. *Microelectronic Engineering* **87**, 810-813 (2010).
10. H. C. Berg, *E. coli in Motion* (Springer Science & Business Media, 2008).
11. F. Alejandro Bonilla, N. Kleinfelter, J. H. Cushman, Microfluidic aspects of adhesive microbial dynamics: A numerical exploration of flow-cell geometry, Brownian dynamics, and sticky boundaries. *Advances in Water Resources* **30**, 1680-1695 (2007).
12. J. M. Swiecicki, O. Sliusarenko, D. B. Weibel, From swimming to swarming: Escherichia coli cell motility in two-dimensions. *Integr Biol (Camb)* **5**, 1490-1494 (2013).
13. C. Acikgoz, M. A. Hempenius, J. Huskens, G. J. Vancso, Polymers in conventional and alternative lithography for the fabrication of nanostructures. *European Polymer Journal* **47**, 2033-2052 (2011).
14. D. Qin, Y. Xia, G. M. Whitesides, Soft lithography for micro- and nanoscale patterning. *Nat Protoc* **5**, 491-502 (2010).
15. M. Gan, J. Su, J. Wang, H. Wu, L. Chen, A scalable microfluidic chip for bacterial suspension culture. *Lab on a Chip* **11**, 4087-4092 (2011).
16. R. Watteaux, R. Stocker, J. R. Taylor, Sensitivity of the rate of nutrient uptake by chemotactic bacteria to physical and biological parameters in a turbulent environment. *J Theor Biol* **387**, 120-135 (2015).
17. C. A. Schneider, W. S. Rasband, K. W. Eliceiri, NIH Image to ImageJ: 25 years of image analysis. *Nat Methods* **9**, 671-675 (2012).
18. E. Meijering, O. Dzyubachyk, I. Smal, Methods for cell and particle tracking. *Methods Enzymol* **504**, 183-200 (2012).

19. K. Bente *et al.*, High-speed motility originates from cooperatively pushing and pulling flagella bundles in bilophotrichous bacteria. *eLife* **9** (2020).
20. H. Shum, E. A. Gaffney, D. J. Smith, Modelling bacterial behaviour close to a no-slip plane boundary: The influence of bacterial geometry. *Proceedings of the Royal Society A: Mathematical, Physical and Engineering Sciences* **466**, 1725-1748 (2010).
21. S. E. Spagnolie, E. Lauga, Hydrodynamics of self-propulsion near a boundary: predictions and accuracy of far-field approximations. *Journal of Fluid Mechanics* **700**, 105-147 (2012).
22. H. Shum, E. A. Gaffney, Hydrodynamic analysis of flagellated bacteria swimming in corners of rectangular channels. *Physical Review E - Statistical, Nonlinear, and Soft Matter Physics* **92** (2015).
23. S. Rivas-Lamelo *et al.*, Magnetotactic bacteria as a new model for P sequestration in the ferruginous Lake Pavin. (2017).
24. P. Dean, M. Maresca, S. Schüller, A. D. Phillips, B. J. P. o. t. N. A. o. S. Kenny, Potent diarrheagenic mechanism mediated by the cooperative action of three enteropathogenic Escherichia coli-injected effector proteins. **103**, 1876-1881 (2006).
25. K. Karamanoli *et al.*, Are leaf glandular trichomes of oregano hospitable habitats for bacterial growth? **38**, 476-485 (2012).
26. L. E. B. Baldotto, F. L. J. C. j. o. m. Olivares, Phylloepiphytic interaction between bacteria and different plant species in a tropical agricultural system. **54**, 918-931 (2008).
27. E. Ringø, R. E. Olsen, T. M. Mayhew, R. J. A. Myklebust, Electron microscopy of the intestinal microflora of fish. **227**, 395-415 (2003).
28. M. M. Baum *et al.*, Characterization of structures in biofilms formed by a Pseudomonas fluorescens isolated from soil. **9**, 103 (2009).
29. I. L. Bagatini *et al.*, Host-specificity and dynamics in bacterial communities associated with bloom-forming freshwater phytoplankton. **9**, e85950 (2014).
